# National and subnational burden of under-5, infant, and neonatal mortality in Ethiopia, 1990–2019: Findings from the Global Burden of Disease Study 2019

**DOI:** 10.1371/journal.pgph.0001471

**Published:** 2023-06-21

**Authors:** Gizachew A. Tessema, Tezera Moshago Berheto, Gavin Pereira, Awoke Misganaw, Yohannes Kinfu, GBD 2019 Ethiopia Child Mortality Collaborators

**Affiliations:** 1 Curtin School of Population Health, Curtin University, Perth, Western Australia, Australia; 2 School of Public Health, University of Adelaide, Adelaide, South Australia, Australia; 3 HIV and TB Research Directorate, Ethiopian Public Health Institute, Addis Ababa, Ethiopia; 4 Centre for Fertility and Health, Norwegian Institute of Public Health, Oslo, Norway; 5 Department of Health Metrics Sciences, School of Medicine, University of Washington, Seattle, Washington, United States of America; 6 National Data Management Center for Health, Ethiopian Public Health Institute, Addis Ababa, Ethiopia; 7 Faculty of Health, University of Canberra, Canberra, Australian Capital Territor, Australia; 8 International Institute of Global Health, United Nations University, Kuala Lumpur, Malaysia; African Population and Health Research Center, KENYA

## Abstract

The under-5 mortality rate is a commonly used indicator of population health and socioeconomic status worldwide. However, as in most low- and middle-income countries settings, deaths among children under-5 and in any age group in Ethiopia remain underreported and fragmented. We aimed to systematically estimate neonatal, infant, and under-5 mortality trends, identify underlying causes, and make subnational (regional and chartered cities) comparisons between 1990 and 2019. We used the Global Burden of Diseases, Injuries, and Risk Factors Study (GBD 2019) to estimate three key under-5 mortality indicators—the probability of death between the date of birth and 28 days (neonatal mortality rate, NMR), the date of birth and 1 year (infant mortality rate, IMR), and the date of birth and 5 years (under-5 mortality rate, U5MR). The causes of death by age groups, sex, and year were estimated using Cause of Death Ensemble modelling (CODEm). Specifically, this involved a multi-stage process that includes a non-linear mixed-effects model, source bias correction, spatiotemporal smoothing, and a Gaussian process regression to synthesise mortality estimates by age, sex, location, and year. In 2019, an estimated 190,173 (95% uncertainty interval 149,789–242,575) under-5 deaths occurred in Ethiopia. Nearly three-quarters (74%) of under-5 deaths in 2019 were within the first year of life, and over half (52%) in the first 28 days. The overall U5MR, IMR, and NMR in the country were estimated to be 52.4 (44.7–62.4), 41.5 (35.2–50.0), and 26.6 (22.6–31.5) deaths per 1000 livebirths, respectively, with substantial variations between administrative regions. Over three-quarters of under-5 deaths in 2019 were due to five leading causes, namely neonatal disorders (40.7%), diarrhoeal diseases (13.2%), lower respiratory infections (10.3%), congenital birth defects (7.0%), and malaria (6.0%). During the same period, neonatal disorders alone accounted for about 76.4% (70.2–79.6) of neonatal and 54.7% (51.9–57.2) of infant deaths in Ethiopia. While all regional states in Ethiopia have experienced a decline in under-5, infant, and neonatal mortality rates in the past three decades, the rate of change was not large enough to meet the targets of the Sustainable Development Goals (SDGs). Inter-regional disparities in under 5 mortality also remain significant, with the biggest differences being in the neonatal period. A concerted effort is required to improve neonatal survival and lessen regional disparity, which may require strengthening essential obstetric and neonatal care services, among others. Our study also highlights the urgent need for primary studies to improve the accuracy of regional estimates in Ethiopia, particularly in pastoralist regions.

## Introduction

Under-5 mortality serves as an essential indicator of socioeconomic status, quality of life, and societal wellbeing [1, 2]. In the past three decades, economic development and the high priority accorded to mothers and children in the Millennium Development Goals (MDGs) have led to a substantial decline in under-5 mortality across the globe [1, 3–6]. However, the burden remains high, particularly in resource-constrained settings such as sub-Saharan Africa (SSA) [7–9]. Current estimates show that although SSA accounts for only a quarter of the global under-5 population, more than half of the nearly 5 million children who died before reaching the age of 5 in 2019 were from the region [1, 10, 11]. Despite achieving the MDG 4 target, Ethiopia is also one of the six countries contributing to more than half of the worldwide child mortality burden in 2019 [1].

The UN Sustainable Development Goal 3 (SDG 3) call to end all preventable deaths and set ambitious targets to reduce neonatal deaths to 12 per 1000 livebirths or less and under-5 deaths to less than 25 per 1000 livebirths by 2030 [12, 13]. With a little less than a decade is remaining before the target date for SDGs, Ethiopia’s reported neonatal mortality rate stood at 33 deaths per 1000 livebirths in 2019 [14]. The neonatal mortality rate in Ethiopia is far higher than the global target for 2019 and exceeds the estimates for SSA, 27 deaths per 1000 livebirths, and the world as a whole, 17 deaths per 1000 livebirths, for the same year [15]. Similarly, the country’s progress in neonatal mortality in the past three decades was relatively slow—less than 2% per annum—compared to post-neonatal under-5 mortality. This experience mirrors the global trend in improvements in neonatal and infant mortality rates—sub-components of under-5 mortality—that lags far behind overall progress in under-5 mortality rates [6–8].

Ethiopia’s 2^nd^ Health Sector Transformation Plan (HSTP-II) targets to reduce the neonatal mortality rate (NMR) to 21, the infant mortality rate (IMR) to 36, and the under-5 mortality rate (U5MR) to 44 deaths per 1000 livebirths by 2025 [16]. Evidence shows that up to 90% of neonatal deaths are avoidable through implementing relatively few interventions: namely, increased access to institutional delivery; access to skilled personnel before, during, and after delivery; increased access to care for low-birthweight neonates; hygienic cord care practice; good thermal care; and on-time initiation and exclusive breastfeeding [7, 14, 17]. However, research on under 5, infant and neonatal mortality in Ethiopia is limited. The few existing studies hardly investigate regional disparities or map underlying causes; when they do so, they are only available for geographically limited areas. As a result, the country lacks the evidence base to monitor the causes and course of under-5 mortality and support its decentralised health system. This study systematically investigates the trends, disparities, and causes of under-5 infant and neonatal deaths in Ethiopia over the past three decades. It compares estimates across the country’s nine administrative regions and two chartered cities. The findings will help measure progress towards the SDGs and track Ethiopia’s national health transformation plan targets.

## Methods

### Overview

The analysis and findings in this paper were produced under the auspices of Ethiopia’s regional burden of disease initiative: a collaborative endeavour between the National Data Management Center for Health (NDMC) at the Ethiopian Public Health Institute (EPHI) and the Institute for Health Metrics and Evaluation (IHME), as part of GBD Study 2019. The EPHI, in collaboration with IHME, gathered all accessible data sources by location for Ethiopia, nine administrative regions, and two chartered cities for analysis as part of the GBD study. NDMC with the support of collaborators and officers from respective regional health bureaus mapped all data sources in the country based on GBD protocol before the data were passed to IHME for analysis as part of the GBD study. GBD 2019 uses the GATHER approach, implements interactive visualisations, and shows final estimates on a dedicated portal, which can be found in GBD Compare [18]. A detailed description of the metrics, data sources, and statistical modelling for child mortality at various geographical levels and causes of death and risk factors has been reported previously [6, 19, 20].

### Setting

Ethiopia is a low-income country located in the eastern part of Africa, with an estimated 115 million people in 2020 [21]. Approximately 80% of the population resides in rural areas where access to health care is limited. About half of the country’s population is under 15, owing to the high fertility rate [16, 22]. Currently, Ethiopia is divided into 11 administrative regions and 2 chartered cities. Out of these, two regions, the Sidama and Southwestern Ethiopia Peoples’ Regional states, were recently carved out of the Southern Nations, Nationalities Peoples Region (SNNPR). However, in this analysis, the two recently established regions were presented as part of SNNPR as there were no separate estimates provided for them. In all, our study covers nine regional states and two chartered cities.

### Data sources

The GBD study is a systematic, scientific effort to quantify the comparative magnitude of health loss due to diseases and injuries by age, sex, and location in the world over time. GBD 2019 provides mortality estimates for 204 countries and subnational territories. Ethiopia is one of the 21 countries globally (and only one of four in Africa) with subnational GBD estimates [23]. For Ethiopia, the GBD 2019 study used multiple data sources, including censuses, verbal autopsy data from Health and Demographic Surveillance System (HDSS) sites, Demographic and Health Surveys (DHS), household surveys, disease registries, health service utilisation, disease notification and Performance Monitoring and Accountability (PMA) surveys, scientific literature, and unpublished reports to estimate under-5 mortality and causes of deaths in Ethiopia [20, 23]. All data were carefully reviewed for quality, including collating input from the GBD collaborator network.

### Modelling and analysis

The GBD estimation process follows the analytical framework for mortality analysis described elsewhere [24]. Briefly, this involves estimating all-cause under-5, infant, and neonatal mortality rates as the probability of dying between birth and 28 days, birth and one year, and birth and five years, per 1000 livebirths, respectively. The estimation followed a multi-stage process that included a non-linear mixed-effects model, source bias correction, spatiotemporal smoothing, and a Gaussian process regression to synthesise mortality estimates by age, sex, location, and year. Three main covariates—complete time series of lag-distributed income (LDI), maternal education, and childhood HIV crude rate—were incorporated into the mixed-effects model. Analogous multi-stage location-year-specific models were used to estimate the sex ratio of under-5 mortality and age- and sex-specific probability of death for neonatal (0–27 days) and infant (under one year) age groups. For estimating the causes of death, GBD 2019 included 369 specific causes of death and injury, broadly grouped hierarchically into four levels that fall under the following three major groups (Level 1): communicable, maternal, neonatal, and nutritional causes; non-communicable diseases; and injuries. These groups were then subdivided into 21 broader categories (Level 2) and subcategories of specific causes (Levels 3 and 4). Causes of death by age groups, sex, and year for other diseases were measured using the Cause of Death Ensemble model (CODEm). A detailed description of CODEm is reported elsewhere [24].

In summary, CODEm tests and selects the best-performing model from a wide range of models, such as mixed-effects linear models and spatiotemporal Gaussian process regression (ST-GPR) models, in predicting mortality rates and cause fractions [24]. The results of all cause-specific models are summed to ensure that the total for all specific diseases and injuries is equal to all-cause mortality. The 95% uncertainty intervals (UIs) were derived for mortality rate estimates, with the 2.5^th^ and 97.5^th^ percentile drawn as the lower and upper bounds. We have provided regional comparisons of overall age-specific mortality and mortality rates by age category and the annual rate of change (AROC) of under-5 mortality by region. We visualised the regional differences of U5MR and rates of changes of U5MR between 1990 and 2019 using QGIS 3.2 geographic information system software. We provided under-5, infant, and neonatal deaths estimates by Level 3 leading causes of death and attributable risk factors. The analysis of causes of death is focused on the top 15 leading Level 3 causes of death, which accounted for over 90% of under-5 deaths in Ethiopia. A complete list of Level 3 causes of death for under 5 children in GBD Study 2019 is provided in a S1 Table.

### Ethical consideration

The GBD 2019 study team obtained ethics approval from the University of Washington Institutional Review Board Committee. The GBD protocol and all data are available online upon request from the EPHI-IHME Office.

## Results

### Number of under-5, infant, and neonatal deaths

In 2019, an estimated 190,173 (95% UI 149,789–242,575) under-5 deaths occurred in Ethiopia, which represented a substantial decline from 462,195 (427,639–499,980) under-5 deaths estimated for 1990. More than half (51.5%) and almost three-quarters (74.2%) of under-5 deaths were neonatal and infant deaths, respectively (Table 1).

**Table 1:** The number of under-5, infant, and neonatal deaths in 1990, 2015, 2019 and rate of change in Ethiopia and administrative regions.

Region	1990	2015	2019	
	Number (95% UI)	Number (95% UI)	Number (95% UI)	ROC (95% UI) 1990 to 2019
**Under-5 deaths**				
Ethiopia	462,195 (427,639–499,980)	229,059 (186,512–276,681)	190,173 (149,789–242,575)	-59 (-68, -47)
Tigray	24,728 (22,657–26,992)	8,232 (6,588–10,088)	6,032 (4,740–7,735)	-76–81, -68)
Afar	5,906 (5,371–6,488)	3,363 (2,706–4,161)	2,660 (2,055–3,462)	-55 (-65, -42)
Amhara	129,161 (119,107–140,323)	49,281 (38,089–61,673)	39,928 (31,589–50,792)	-69 (-76, -61)
Oromia	153,902 (141860–167618)	91,423 (75,580–109,583)	76,573 (60,612–96,984)	-50 (-61, -36)
Somali	18,974 (17,318–20,738)	19,833 (16,175–23,860)	17,751 (14,194–22,340)	-6 (-26, 18)
Benishangul Gumuz	5,752 (5,142–6,457)	3,251 (2,560–4,045)	2,722 (2,104–3,528)	-53 (-64, -38)
SNNPR*	110,622 (101,110–120,512)	50,313 (40,507–61,544)	42,086 (32,800–54,521)	-62 (-71, -51)
Gambella	1,765 (1,605–1,940)	677 (534–843)	508 (392–666)	-71 (-78, -62)
Harari	1,245 (1119–1384)	429 (340–529)	329 (256–426)	-74 (-80, -66)
Dire Dawa	2,542 (2,272–2,838)	822 (651–1015)	627 (487–814)	-75 (-81, -68)
Addis Ababa	7,598 (6,526–8,842)	1,436 (1,042–1,909)	957 (694–1320)	-87 (-91, -82)
**Infant deaths**				
Ethiopia	275,326 (252,240–300,220)	164,266 (131,719–200,202)	141,063 (111,137–180,143)	-49 (-60, -34)
Tigray	14,947 (13,615–16,398)	6,259 (4,980–7,778)	4,712 (3,695–6,056)	-68 (-76, -59)
Afar	3,820 (3,443–4255)	2,466 (1,951–3,093)	2,007 (1,554–2,617)	-47 (-60, -32)
Amhara	75,163 (68,765–81,891)	34,771 (26,793–43,922)	29,253 (23,071–37,365)	-61 (-70, -50)
Oromia	93,228 (85,392–102,207)	66,015 (53,976–79891)	57,235 (45,316–72,755)	-39 (-52, -22)
Somali	12,266 (11,183–13,409)	13,874 (11,218–16,941)	12,709 (10,155–16,012)	4 (, -18, 32)
Benishangul Gumuz	3,219 (2,837–3,664)	2,202 (1,751–2,707)	1,916 (1,479–2,502)	-40 (-55, -22)
SNNPR*	64,343 (58,525–70,865)	36,119 (28,806–44,762)	31,363 (24,432–40,696)	-51 (-62, -37)
Gambella	1,022 (921–1,140)	508 (398–641)	397 (305–520)	-61 (-70, -49)
Harari	750 (665–848)	314 (247–393)	248 (192–323)	-67 (-75, -57)
Dire Dawa	1,485 (1,310–1,689)	601 (470–751)	474 (367–616)	-68 (-75, -58)
Addis Ababa	5,082 (4,297–6,023)	1,136 (823–1,512)	747 (544–1,033)	-85 (-89, -79)
**Neonatal deaths**				
Ethiopia	136, 288 (124,637–149,504)	11,0340 (88,324–13,4736)	97, 920 (77,161–125,717)	-28 (-44, -7)
Tigray	7,438 (6,764–8,174)	4,543 (3,608–5,655)	3,541 (2,774–4,554)	-52 (-63, -38)
Afar	1,953 (1,757–2,179)	1,722 (1,364–2,163)	1,449 (1,122–1,887)	-26 (-43, -4)
Amhara	36,820 (33,568–40,169)	22,951 (17,674–29,016)	19,996 (15,743–25,585)	-46 (-58, -30)
Oromia	46,233 (42,185–50,903)	44,522 (36,350–53,922)	39,965 (31,649–51,015)	-14 (-33, 11)
Somali	6,359 (5,787–6,958)	9,132 (7,370–11,154)	8,530 (6,813–10,772)	34 (6, 70)
Benishangul Gumuz	1,555 (1,366–1,774)	1,396 (1,110–1,721)	1,247 (962–1,630)	-20 (-39, 5)
SNNPR*	31,574 (28,653–34,889)	24,240 (19,309–30,101)	21,833 (17,011–28,373)	-31 (-47, -10)
Gambella	499 (448–559)	355 (278–448)	290 (223–380)	-42 (-55, -24)
Harari	371 (328–420)	213 (167–266)	173 (134–226)	-53 (-64, -40)
Dire Dawa	721 (636–824)	405 (316–508)	331 (257–430)	-54 (-64, -40)
Addis Ababa	2,766 (2,326–3,285)	860 (623–1146)	565 (412–782)	-80 (-85, -71)

ROC- Rate of change. UI—Uncertainty Interval. SNNPR-Southern Nations Nationality and People Region.

*SNNPR comprises Sidama Region and Southwest Ethiopia Peoples’ Region

### Under-5, infant, and neonatal mortality rates

**Fig 1:**
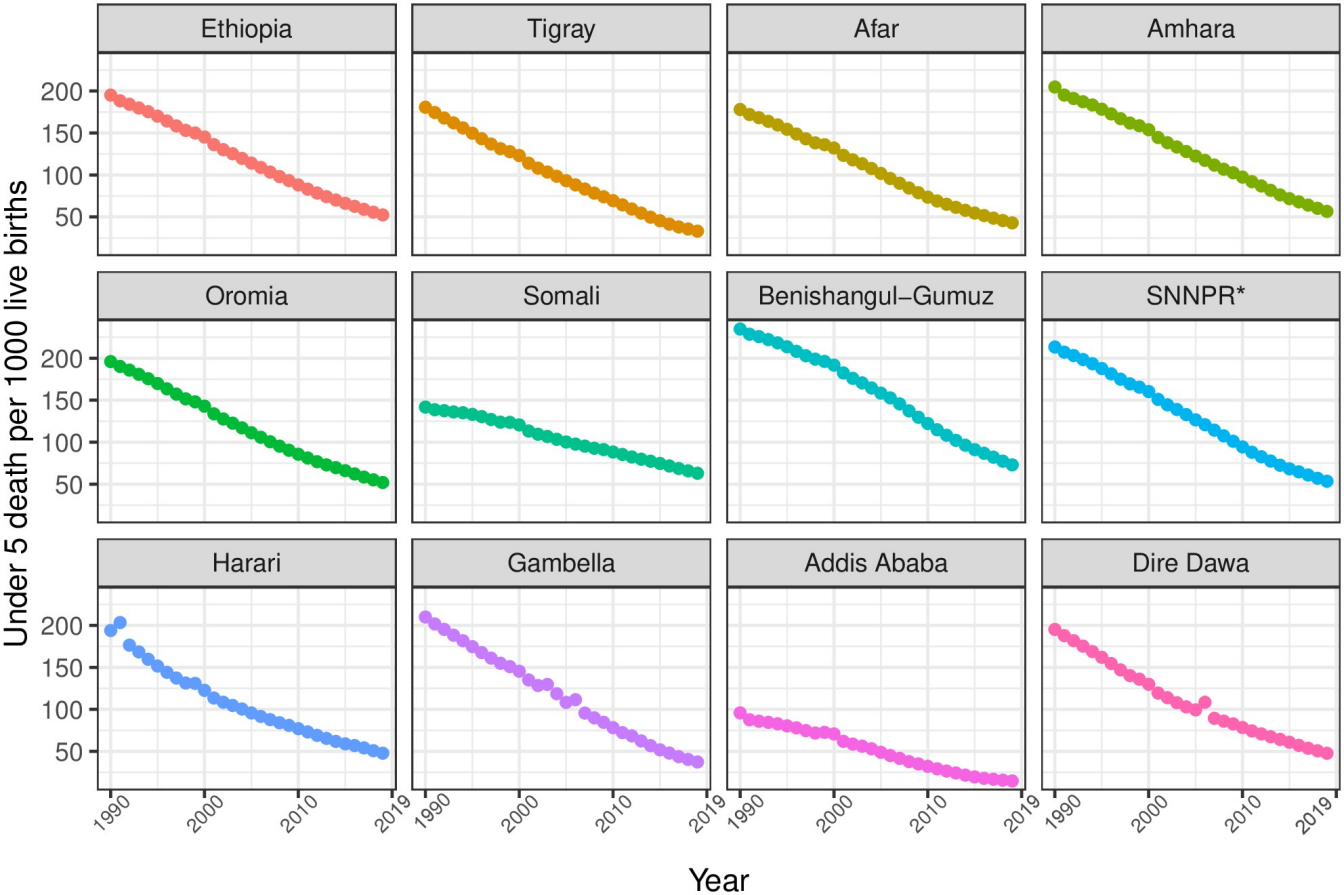
Trends in under-5 mortality rate in Ethiopia and its regional states during 1990–2019. *SNNPR comprises Sidama Region and Southwest Ethiopia Peoples’ Region.

**Fig 2:**
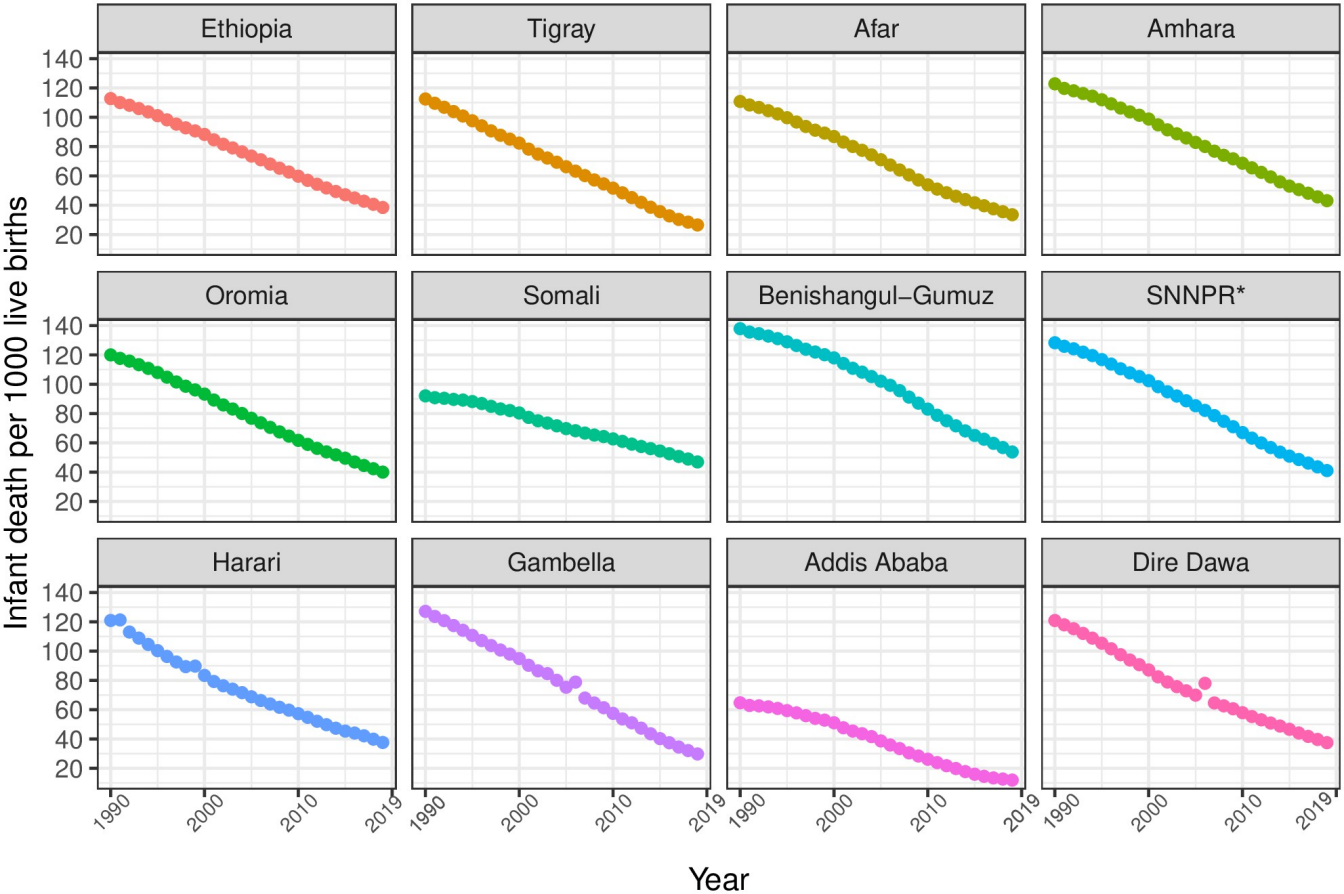
Trends in infant mortality rate in Ethiopia and its regional states during 1990–2019. *SNNPR comprises Sidama Region and Southwest Ethiopia Peoples’ Region.

**Fig 3:**
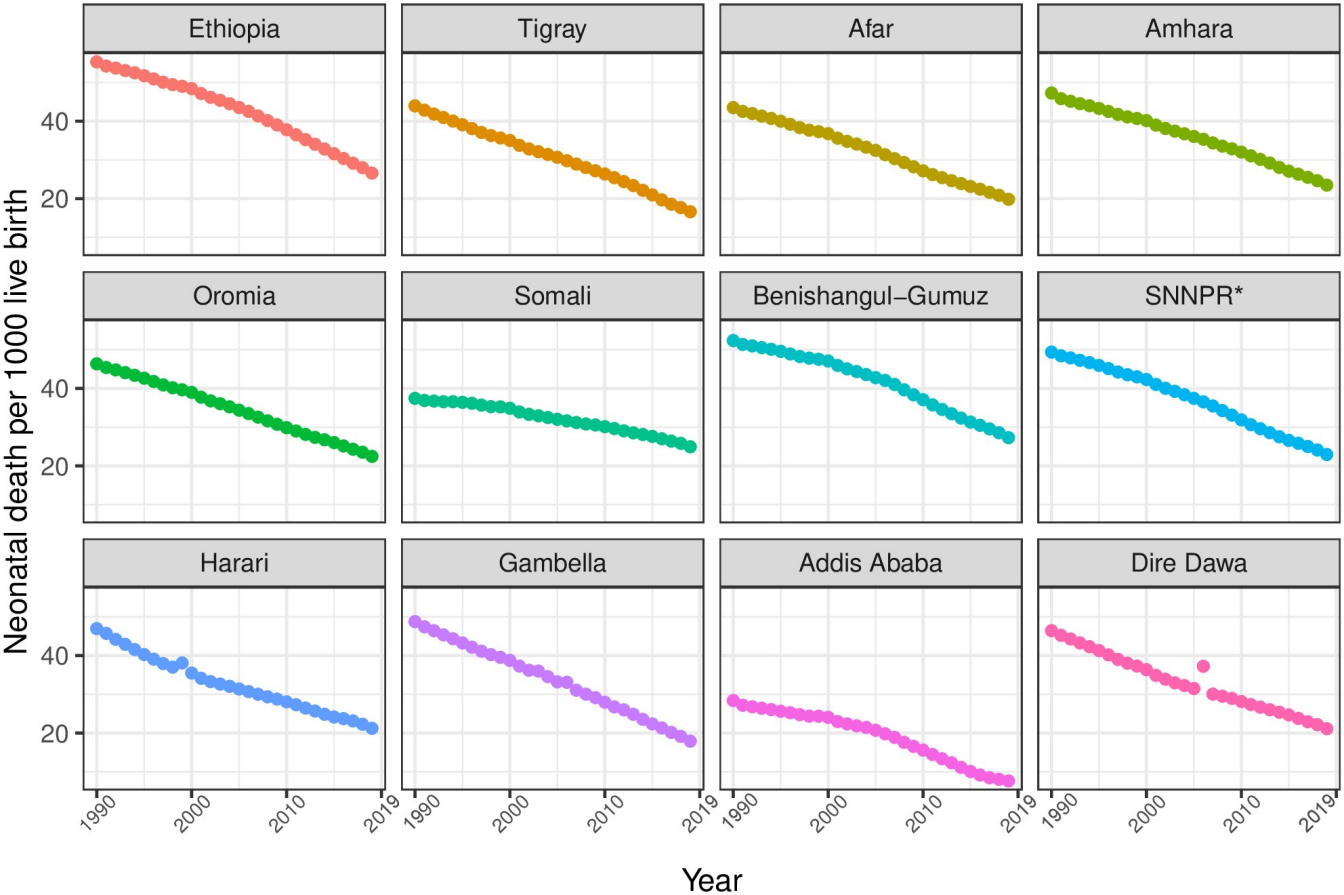
Trends in NMR in Ethiopia and its administrative regions during 1990–2019. *SNNPR comprises Sidama Region and Southwest Ethiopia Peoples’ Region.

In Ethiopia, between 1990 and 2019, U5MR, IMR, and NMR decreased from 195 (95% UI 181.1–209.4) to 52.4 (44.7–62.4), from 128.8 (118.3–139.7) to 41.5 (35.2–50.0), and from 55.3 (51.1–59.5) to 26.6 (22.6–31.5) per 1000 livebirths, respectively. In 2019, U5MR across regions varied widely, ranging from 14.8 (12.4–17.8) in Addis Ababa to 62.9 (53.6–74.9) in Benishangul-Gumuz; IMR varied from 12.2 (10.2–14.8) in Addis Ababa to 57.1 (48.2–68.7) in Benishangul-Gumuz. In contrast, four regions—Amhara, Benishangul-Gumuz, Somali, and SNNPR—observed rates higher than the national rates. For the same period, differences in NMR ranged from 7.6 (6.4–9.3) in Addis Ababa to 27.3 (23.1–32.7) in Benishangul-Gumuz among regions, and except for Benishangul-Gumuz, most regions observed slightly lower rates than the national NMR (Table 2, Figs 1–3).

**Table 2:** The rate of under-5, infant, and neonatal deaths, and rate of change in 1990, 2000, 2015, 2019 in Ethiopia and administrative regions.

Country/ region	Death rate per 1,000 livebirths (95%UI)				%AROC (95% UI)			
	1990	2000	2015	2019	1990–2019	1990–2000	2000–2015	2015–2019
**U5MR**								
Ethiopia	195 (181.1, 209.4)	145.2 (134.2, 157.6)	66.2 (57.4, 75.9)	52.4 (44.7, 62.4)	-4.5 (-4.8, -4.2)	-2.9 (-3, -2.8)	-5.2 (-5.7, -4.9)	-5.8 (-6.3, -4.9)
Tigray	180.7 (165.5, 196.6)	123.1 (111.7, 134.8)	45.4 (38.9, 52.4)	33.1 (28.1, 39.6)	-5.9 (-6.1, -5.5)	-3.8 (-3.9, -3.8)	-6.6 (-7, -6.3)	-7.9 (-8.1, -7)
Afar	177.9 (162.9, 193.7)	132.1 (120.5, 143.5)	54.7 (46.4, 63.7)	42.9 (36.1, 51.9)	-4.9 (-5.2, -4.5)	-3 (-3, -3)	-5.9 (-6.4, -5.4)	-6.1 (-6.3, -5.1)
Amhara	204.7 (190.6, 220.4)	153.8 (141.3, 168.3)	71.8 (59.1, 86.3)	56.6 (48.4, 67.2)	-4.4 (-4.7, -4.1)	-2.9 (-3, -2.7)	-5.1 (-5.8, -4.5)	-5.9 (-5, -6.3)
Oromia	195.9 (180.3, 212.3)	145.2 (134.2,157.6)	66 (57.9, 75.4)	51.9 (44.3, 61.8)	-4.6 (-4.8, -4.3)	-3.2 (-3.1, -3.1)	-5.1 (-5.5, -4.8)	-6 (-6.7, -5)
Somali	141.8 (130.2, 153.7)	120.5 (109.9,132.3)	74.6 (63.9, 85.6)	62.9 (53.6, 74.9)	-4 (-4.3, -3.8)	-2 (-2.5, -1.7)	-5 (-5.2, -4.8)	-5.6 (-5, -5.1)
Benishangul- Gumuz	234.7 (214.1, 258.1)	191.7 (166.8, 218.4)	91.1 (76.2, 106.3)	72.9 (62.4, 86.6)	-2.8 (-3.1, -2.5)	-1.6 (-1.7, -1.5)	-3.2 (-3.6, -2.9)	-4.3 (-4.4, -3.3)
SNNPR*	213.3 (196.3, 231.7)	160.2 (145.9, 179.8)	68.3 (59, 78.3)	53.4 (45.6, 63.5)	-4.8 (-5, -4.5)	-2.9 (-3, -2.5)	-5.7 (-6, -5.5)	-6.2 (-6.4, -5.2)
Gambella	210 (193, 228.1)	145.3 (132.7, 158.9)	51.8 (44.7, 59.7)	37.3 (31.6, 44.6)	-6 (-6.2, -5.6)	-3.7 (-3.7, -3.6)	-6.9 (-7.3, -6.5)	-8.2 (-8.7, -7.3)
Harari	193.9 (177.4, 210.9)	122.7 (105.8, 142.1)	58.9 (50.9, 67.7)	47.7 (40.7, 56.9)	-4.8 (-5.1, -4.5)	-4.6 (-5.2, -3.9)	-4.9 (-4.9, -4.9)	-5.3 (-5.6, -4.3)
Dire Dawa	195.1 (178.5, 212.3)	129.8 (112.5, 150.3)	60.8 (52.4, 70)	47.7 (40.6, 56.9)	-4.9 (-5.1, -4.5)	-4.1 (-4.6, -3.5)	-5.1 (-5.1, -5.1)	-6.1 (-6.4, -5.2)
Addis Ababa	95.8 (86.7,105.9)	70.7 (59.9,82.8)	19.5 (16,23.4)	14.8 (12.4, 17.8)	-6.4 (-6.7, -6.1)	-3 (-3.7, -2.5)	-8.6 (-8.8, -8.4)	-6.9 (-6.4, -6.8)
**IMR**								
Ethiopia	128.8 (118.3, 139.7)	100.3 (92.3, 109.4)	51.5 (44.2, 59.3)	41.6 (35.2, 50)	-3.9 (-3.5, -4.2)	-2.5 (-2.5, -2.4)	-4.4 (-4.9, -4.1)	-5.3 (-5.7, -4.3)
Tigray	123.5 (111.4, 135.8)	88.8 (80.0, 97.8)	37.2 (31.6, 43.2)	27.4 (23.1, 33.1)	-3.9 (-4.2, -3.5)	-3.3 (-3.3, -3.3)	-5.8 (-6.2, -5.4)	-7.6 (-7.8, -6.7)
Afar	121.4 (109.9, 133.8)	93.4 (84.7, 102.2)	43.7 (36.8, 51.5)	34.9 (29.1, 42.6)	-5.2 (-5.4, -4.9)	-2.6 (-2.6, -2.7)	-5.1 (-5.6, -4.6)	-5.6 (-5.9, -4.7)
Amhara	136 (125, 149)	107.3 (97.8, 118.2)	56.2 (45.8, 68.2)	45.3 (38.3, 54.5)	-4.3 (-4.6, -3.9)	-2.4 (-2.5, -2.3)	-4.3 (-5.1, -3.7)	-5.4 (-4.5, -5.6)
Oromia	132.6 (120.5, 145.9)	100.8 (92.6, 110.7)	52.3 (45.4, 60.1)	41.9 (35.4, 50.4)	-3.8 (-4.1, -3.5)	-2.7 (-2.6, -2.8)	-4.4 (-4.8, -4.1)	-5.5 (-6.2, -4.4)
Somali	99.4 (90.8, 108.9)	86.4 (78.2, 95.2)	57.8 (49, 66.9)	49.5 (41.8, 59.6)	-4 (-4.2, -3.7)	-1.7 (-2.2, -1.3)	-4.2 (-4.4, -4.1)	-4.9 (-4.5, -4.4)
Benishangul- Gumuz	154.7 (138.9, 173.1)	130.2 (111.1, 151.5)	69.5 (57.6, 81.9)	57.1 (48.2, 68.7)	-3.4 (-3.6, -3.2)	-1.4 (-1.5, -1.3)	-2.7 (-3.1, -2.4)	-3.9 (-4, -2.9)
SNNPR*	142.7 (129.6, 156.8)	111.4 (100.8, 123.6)	53.9 (46.2, 62.4)	43.1 (36.4, 51.8)	-2.4 (-2.7, -2.1)	-2.5 (-2.5, -2.4)	-4.8 (-5.2, -4.6)	-5.6 (-6, -4.7)
Gambella	141.3 (128.1, 155.1)	102.7 (93.3, 113.4)	42.2 (36.1, 49.1)	30.9 (26.1, 37.3)	-4.1 (-4.4, -3.8)	-3.2 (-3.2, -3.1)	-5.9 (-6.3, -5.6)	-7.8 (-8.1, -6.9)
Harari	133.6 (120.6, 147.7)	90 (76.8, 105.4)	47.9 (41, 55.6)	39.4 (33.3, 47.4)	-5.2 (-5.5, -4.9)	-4 (-4.5, -3.4)	-4.2 (-4.2, -4.3)	-4.9 (-5.2, -4)
Dire Dawa	133.6 (120.4, 147.7)	94.2 (81.0, 109.9)	16.3 (13.3, 19.7)	39.2 (33.1, 47.3)	-4.2 (-4.4, -3.9)	-3.5 (-4, -3)	-4.3 (-4.4, -4.3)	-5.7 (-6, -4.8)
Addis Ababa	69.2 (61.6, 77.6)	54.3 (45, 64.8)	14.8 (12.1, 17.8)	12.2 (10.2, 14.8)	-4.2 (-4.5, -3.9)	-2.4 (-3.1, -1.8)	-8 (-8.1, -7.9)	-7.2 (-6.6, -7.1)
**NMR**								
Ethiopia	55.3 (51.1, 59.5)	48.4 (44.4, 53.2)	31.6 (27.3, 36.7)	26.6 (22.6, 31.6)	-2.5 (-2.8, -2.2)	-1.3 (-1.4, -1.1)	-2.8 (-3.2, -2.5)	-4.3 (-4.7, -3.7)
Tigray	44 (40, 48.1)	35 (31.6, 38.4)	22.4 (19.2, 25.9)	16.6 (14.1, 20)	-3.4 (-3.6, -3)	-2.3 (-2.4, -2.3)	-3.4 (-3.8, -3.1)	-5.9 (-6, -4.9)
Afar	43.5 (39.7, 47.6)	36.8 (33.4, 40.1)	24.1 (20.8, 28)	19.8 (16.6, 24.1)	-2.7 (-3, -2.3)	-1.7 (-1.7, -1.7)	-3.1 (-3.6, -2.6)	-3.9 (-4.2, -3)
Amhara	47.3 (43.7, 51.3)	40.2 (36.9, 43.9)	26 (22.7, 29.8)	23.5 (19.9, 28.1)	-2.4 (-2.7, -2.1)	-1.6 (-1.7, -1.6)	-2.6 (-3.4, -2)	-3.6 (-2.7, -3.7)
Oromia	46.3 (42.5, 50.5)	39 (35.9, 42.5)	27.6 (23.5, 32)	22.4 (19, 26.9)	-2.5 (-2.8, -2.2)	-1.7 (-1.7, -1.7)	-2.7 (-3.1, -2.4)	-3.7 (-4.4, -2.6)
Somali	37.4 (34.3, 40.8)	34.9 (31.6, 38.3)	26.6 (22.8, 30.7)	24.9 (21.1, 30)	-2.2 (-2.5, -2)	-1 (-1.5, -0.7)	-2.7 (-3, -2.6)	-3.4 (-3, -3)
Benishangul- Gumuz	52.3 (47.4, 57.8)	47.1 (40.7, 54.1)	21 (17.9, 24.3)	27.3 (23.1, 32.7)	-1.4 (-1.7, -1.1)	-0.7 (-0.8, -0.6)	-1.6 (-2, -1.2)	-2.6 (-2.7, -1.6)
SNNPR*	48.8 (44.6, 53.3)	42.3 (38.5, 46.5)	7.6 (6.4, 9.3)	17.9 (15.1, 21.5)	-2.7 (-2.9, -2.3)	-1.6 (-1.6, -1.5)	-3.1 (-3.5, -2.8)	-3.7 (-4, -2.8)
Gambella	47 (42.8, 51.6)	38.8 (35.3, 42.6)	19.8 (16.6, 24.1)	21.2 (18, 25.5)	-3.5 (-3.7, -3.1)	-2.3 (-2.3, -2.2)	-3.7 (-4.1, -3.3)	-5.6 (-6, -4.7)
Harari	49.4 (45.1, 53.9)	35.5 (30.4, 41.2)	23.5 (19.9, 28.1)	22.9 (19.4, 27.5)	-2.7 (-3, -2.4)	-2.8 (-3.4, -2.3)	-2.6 (-2.5, -2.6)	-3.2 (-3.6, -2.3)
Dire Dawa	46.4 (42.1, 51)	36.4 (31.4, 42.3)	27.3 (23.1, 32.7)	21.1 (17.9, 25.4)	-2.7 (-2.9, -2.4)	-2.4 (-2.9, -1.9)	-2.6 (-2.6, -2.6)	-3.9 (-4.2, -3.1)
Addis Ababa	28.4 (25.4, 31.7)	24.1 (20.1, 28.5)	21.1 (17.9, 25.4)	7.6 (6.4, 9.3)	-4.5 (-4.8, -4.2)	-1.6 (-2.3, -1.1)	-5.8 (-5.9, -5.7)	-7.1 (-6.5, -6.6)

U5MR—Under 5 mortality Rate, IMR- Infant Mortality Rate, NMR- Neonatal Mortality Rate, UI—Uncertainty Interval. AROC—Annual Rate of Change, SNNPR-Southern Nations Nationality and People Region.

*SNNPR comprises Sidama Region and Southwest Ethiopia Peoples’ Region.

**Fig 4:**
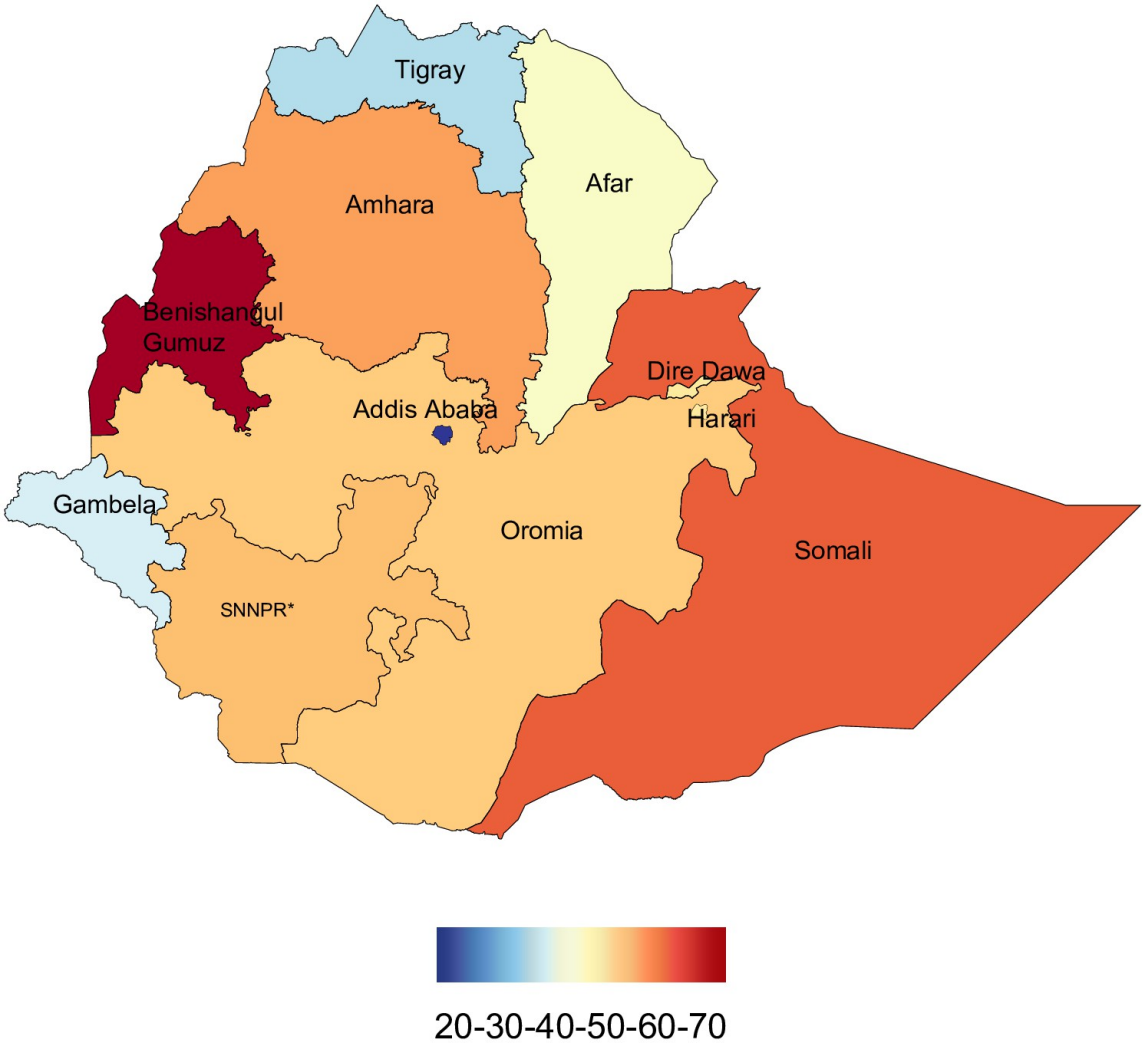
Under-5 mortality rates per 1,000 live births in its administrative regions of Ethiopia in 2019. *SNNPR comprises Sidama Region and Southwest Ethiopia Peoples’ Region. The basemap for the shapefile was sourced from: https://data.humdata.org/m/dataset/cod-ab-eth.

**Fig 5:**
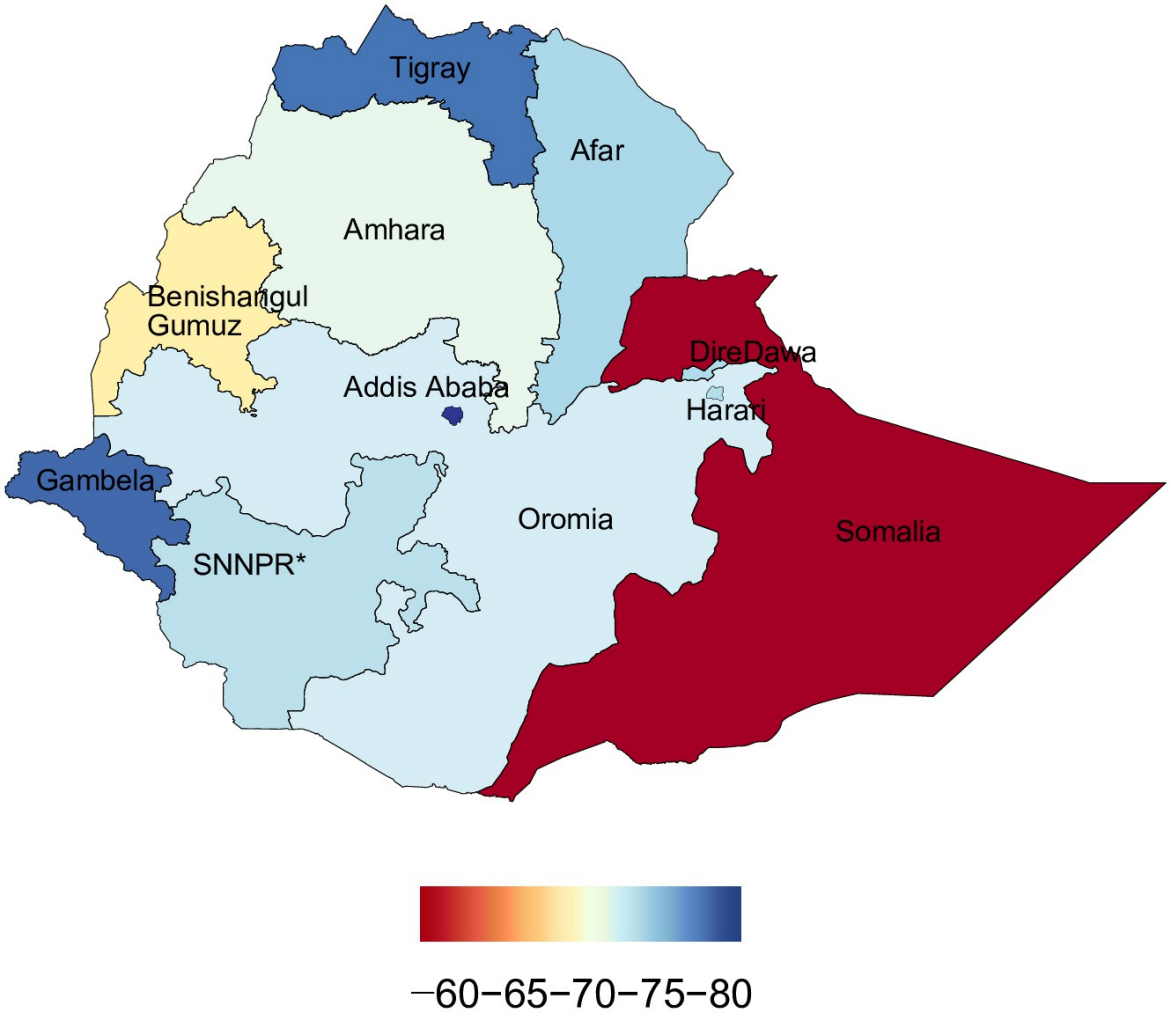
Under-5 mortality percent change in administrative regions of Ethiopia during 1990–2019. *SNNPR comprises Sidama Region and Southwest Ethiopia Peoples’ Region. The basemap for the shapefile was sourced from: https://data.humdata.org/m/dataset/cod-ab-eth.

From 1990 to 2019, U5MR, IMR, and NMR decreased nationally, but differences in average rates of change (AROC) were substantial across regions. The AROC for U5MR ranged from 2.8% (Benishangul-Gumuz) to 6.4% (Addis Ababa). For IMR, the AROC ranged from 2.4% (SNNPR) to 5.2% (Afar and Harari). Similarly, for NMR, the AROC ranged from 1.4% (Somali) to 4.5% (Addis Ababa). For all three rates, the AROC was relatively lower before 2000 and showed improvement from 2000 to 2015, a momentum maintained until 2019 (Table 2, Figs 4 and 5).

U5MR, IMR, and NMR were higher in males than females, although there was no statistically significant difference at the national level (S2 Table).

### Leading causes of under-5, infant, and neonatal deaths in 2019

**Fig 6:**
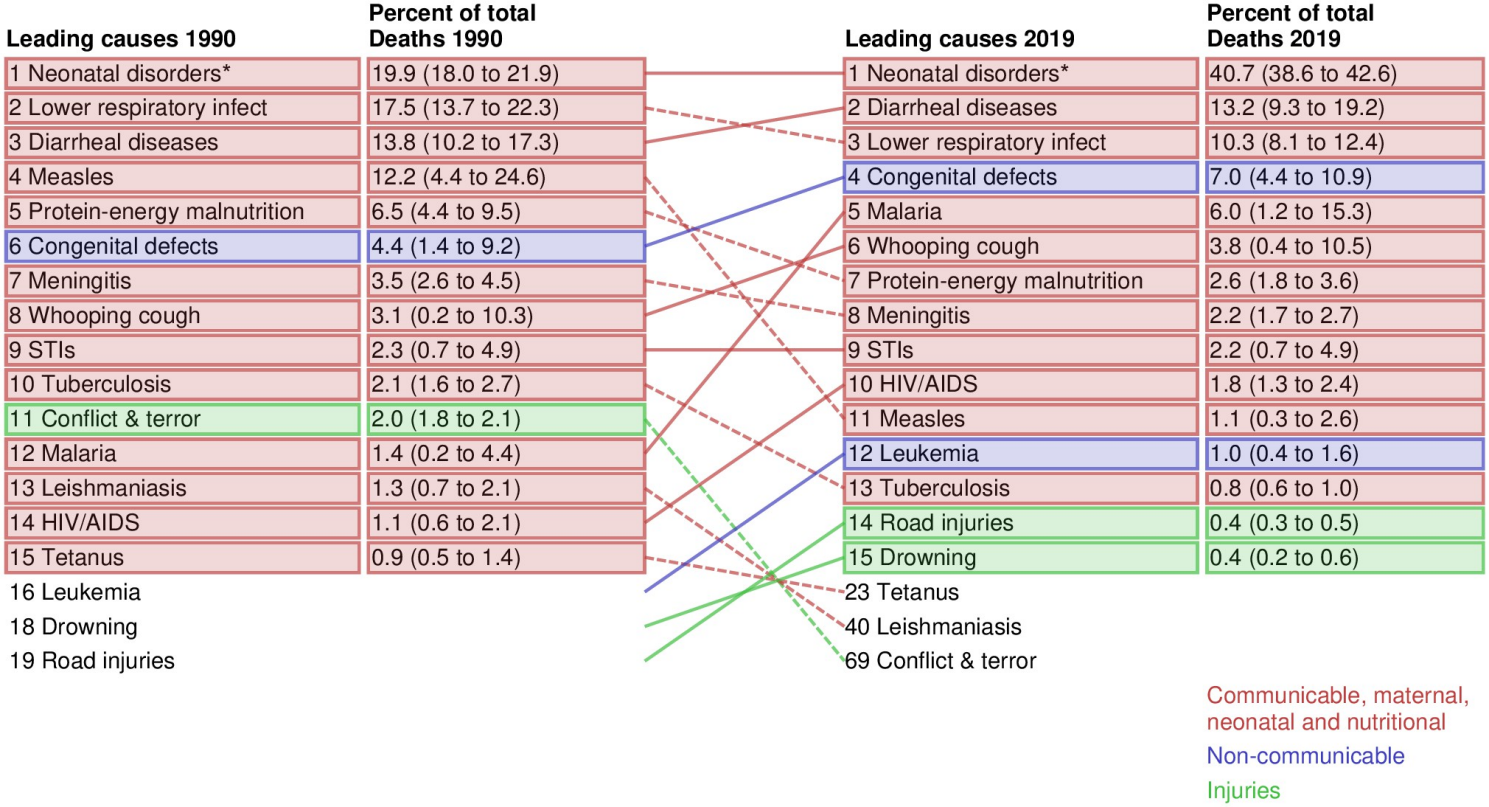
Level 3 leading causes of deaths for under-5 mortality in Ethiopia in 1990 and 2019. *Neonatal disorders include pre-term births, neonatal encephalopathy due to birth asphyxia and trauma, neonatal sepsis and other neonatal infectious, hemolytic diseases and other neonatal jaundice, and other neonatal disorders. STI- Sexual Transmitted Infections. HIV–Human Immunodeficiency virus. AIDS—Acquired Immunodeficiency Diseases. UI- Uncertainty interval.

Neonatal disorders such as preterm birth complications, neonatal encephalopathy and birth trauma, and neonatal sepsis and other infections (40.7%, 95% UI 38.6–42.6), diarrhoeal diseases (13.2%, 9.3–19.2), lower respiratory infections (10.3%, 8.1–12.4), congenital birth defects (7.0%, 4.4–10.9), and malaria (6.0%, 1.2–15.3) were the top five Level 3 causes of deaths in 2019 (Fig 6). During the same period, neonatal disorders alone accounted for about 76.4% (70.2–79.6) of neonatal deaths and 54.7% (51.9–57.2) of infant deaths in Ethiopia (S1 and S2 Figs).

### Under-5, infant, and neonatal deaths attributable to risk factors

In 2019, 61.6% (95% UI 55.1–67.8) of under-5, 68.0% (62.9–72.7) of infant, and 75.8% (71.2–79.4) of neonatal deaths that occurred in Ethiopia were explained by a number of risk factors included in the GBD 2019 study. Child and maternal malnutrition, unsafe water and sanitation, air pollution, and lack of handwashing facilities were the leading risk factors for under-5, infant, and neonatal deaths. While child and maternal malnutrition alone contributed 57.2% (51.3–62.8) of under-5 deaths and 63.6% (59.0–68.1) of infant deaths, low birthweight and short gestation contributed 71.1% (67.2–74.6) of neonatal deaths. The attributable risk factors for under-5 deaths were consistent across administrative regions (S3–S5 Tables).

## Discussion

The study investigated trends in the burden, causes, and risk factors of under-5, infant, and neonatal death in Ethiopia and its 9 administrative regions and 2 chartered cities over the past three decades. In 2019, U5MR, IMR, and NMR were estimated to be 52.4, 41.5, and 26.6 per 1000 livebirths, respectively. The leading causes of under-5 mortality were neonatal disorders, diarrhoeal diseases, lower respiratory infections, congenital birth defects, and malaria. Undernutrition, air pollution, and lack of hygiene and sanitation were the attributable risk factors for most under-5 deaths.

The national estimates in this study are in broad agreement with estimates from the UN Inter-agency Group for Child Mortality (IGME), which were 50.8, 36.6, and 27.6 deaths per 1000 livebirths for U5MR, IMR, and NMR, respectively [25, 26]. However, the U5MR level from our study was far higher than reported in another small-scale local study in Northern Ethiopia (35.6 deaths per 1000 livebirths) [27], but slightly lower than the level reported in a small study from eastern Ethiopia (66.4 per 1000 live births) [28]. We found no significant difference with the level reported in the 2019 small-scale Ethiopian Mini DHS (EMDHS) (59 death per 1000 livebirths) [14] while the data was not included in the GBD 2019 study. Similarly, the neonatal death rate in the current study is lower than but not significant in the same survey reported, which was 33 per 1000 livebirths [14], and data from a localised study in western Ethiopia which reported 35.5 per 1000 livebirths [29]. On the other hand, our national estimates are much lower than those reported in a study in northwest Ethiopia, 43.8 neonatal deaths per 1000 livebirths [30]. However, a study from eastern Ethiopia showed lower rates of neonatal mortality (20.3 deaths per 1000 livebirths) than ours [31]. Besides, we observed differences at regional levels. While our study estimated U5MR for Gambella was 37.3 deaths per 1,000 live births, the corresponding estimates were 86 per 1,000 livebirths in the 2019 EMDHS [14] and 56.5 deaths per 1,000 livebirths in the UN IGME [25]. Similarly, differences were observed in the regional estimates for Addis Ababa (14.8 per 1000 live births in our estimate vs 28.8 per 1,000 live births in the UN IGME estimate) and Afar (74.3 per 1000 live births in our estimate vs 42.9 per 1,000 live births in UN IGME estimate) [25]. The differences in mortality from other studies might be attributable to study design and period of data collection. For example, unlike the GBD methodology that estimated under-5 mortality for a specific year, EDHS studies reported cumulative average for under-5 mortality rate which date as far back as 5 to 10 years ago for national and regional estimates. Moreover, although both the GBD and the UN IGME estimates relied on multiple data sources such as DHS surveys and census data, the latter did not use data from the HDSS that could provide relatively accurate information for estimating U5MR. Moreover, unlike the GBD estimates that used covariates; based on lagged distributed income per capita, mean years of education for women of reproductive age (15–49 years), HIV death rate due to HIV in age groups 0–4 to estimate mortality [24], the UN IGME models did not use covariates but fits Bayesian B-splines bias-adjusted model through observed data on mortality [26]. However, it needs to be noted that given both estimates are model-based, it is likely that the estimates could be affected by the available data, population coverage, data collection period, and the performance of the underlying statistical techniques [27].

In line with the findings from other studies [32], we observed substantial inter-regional disparities in under-5 mortality in Ethiopia, which may be linked to prevailing sociodemographic and structural factors in the country [16, 22, 25]. According to a government report, there are unjust differences in socioeconomic status and core development infrastructures, such as the distribution of educational and health facilities and road and communication networks [14]. In our study, the Somali and Benishangul-Gumuz regions, which exhibited higher levels of under-5 mortality rates than other regions, are, for example, predominantly pastoralist and rural, where road infrastructure and access to essential maternal and child health services [22], including vital interventions to reduce vaccine-preventable childhood diseases, are very limited [33, 34]. This is further supported by a recent study that demonstrated the presence of inequalities in immunisation coverage across administrative regions in Ethiopia [35, 36]. Despite this, the relatively lower estimates of U5MR for Gambella are unexpected and may call for further region-specific empirical data.

Ethiopia’s success in improving under-5 mortality could be linked to global initiatives on maternal and child health interventions that followed the adoption of the MDGs [12, 13, 16]. Additionally, increased health expenditure and the introduction of community health programmes through health extension workers and the Women’s Development Army [37–40] have brought services closer to communities. This, in turn, improved immunisation coverage, access to healthcare services, and sanitation in rural and hard-to-reach communities could be the cornerstones to under-5 mortality reductions.

Current under-5 and neonatal estimates in Ethiopia are almost twice and three times higher than the SDG targets, which aims to reduce under-5 and neonatal mortality to 25 and 12 deaths per 1000 livebirths [13]. The NMR in this study is also 60% higher than the current global average (17 deaths per 1000 livebirths) [1]. Additionally, the slow rate of change in neonatal and infant mortality compared to those aged 1–4 years means that without a renewed newborn-focused intervention, Ethiopia is likely to miss the SDG target [6, 9, 14, 41].

Studies have shown that as the share of neonatal to under-5 mortality increases, health interventions should shift toward improving access to skilled newborn care and expanding institutional delivery and efficient referral services for complicated pregnancies [9]. However, such services are limited in Ethiopia, which explains the slow progress in NMR nationally and the relatively faster decline in more urbanised regions, where such services are broadly accessible to the population. For example, whilst the overall reduction in neonatal deaths in the country was 28% between 1990 and 2019, it was higher in Dire Dawa (80%) and Addis Ababa (54%) [6, 14, 41].

The existing evidence mapped that the causes of under-5 deaths in resource-poor settings are preventable [33, 42–44]. In Ethiopia, over three-quarters of under-5 deaths are due to the following five leading causes: neonatal disorders, diarrhoeal diseases, lower respiratory infections, congenital birth defects, and malaria. For example, neonatal disorders such as preterm birth complications, neonatal encephalopathy and birth trauma, and neonatal sepsis and other infections contributed to approximately 40% of the under-5 deaths. In addition, diseases such as whooping cough, protein-energy malnutrition, and meningitis are also among the major killers of under-5 children, despite a significant reduction in vaccine-preventable conditions following the MDG era [15, 41]. Moreover, according to the World Health Organization, almost all neonatal deaths are due to diarrhoeal disease, protein-energy malnutrition, and neonatal preterm births, while some nutritional risks are associated with adolescent pregnancy and closely spaced conceptions [42, 45]. Implementation of public health and clinical interventions, such as improving access to skilled newborn care, expanding institutional delivery and efficient referral services for complicated pregnancies, providing safe water and sanitation, and promoting handwashing and breastfeeding practices, could facilitate the SDG targets for neonatal and under-5 deaths.

However, many underlying conditions hinder the country’s effort to achieve the SDG targets. First, the advent of the COVID-19 pandemic, which diverted global attention and resources away from earlier global initiatives, is likely to adversely impact health expenditure and other investments on proximate social determinants of health. Cold supply chain disruptions and diversion of essential health resources (such as health facilities and health workers) to meet COVID-19-related needs are also expected to impact progress in child health adversely. Although COVID-19-related mortality is lower for under-5 children [46], the pandemic has hugely affected access to general and essential health services, including immunisation [47, 48]. War generates population displacement; it destroys health system infrastructures, interrupts health service delivery, diverts political leadership’s attention, drains scarce resources away from essential services, and leads to the collapse of the public health system [49, 50]. Particularly, the recent civil war in Ethiopia could worsen inequities in access to services and health outcomes. In light of this notion, our study may serve as a benchmark to compare the impacts of these catastrophic events in Ethiopia and other countries in similar predicaments. Specifically, in the case of Ethiopia, the conflicts and displacements in Tigray, Afar, Amhara, and parts of Western Oromia regions are expected to have far-reaching implications on child health and are likely to reverse decades of progress made in these regions. Other parts of the country are also expected to be affected, albeit to a lesser degree, because of the potential diversion of resources and government attention away from social development.

This study provided the best available comprehensive evidence on under-5 mortality at the national and regional level in Ethiopia for over three decades, allowing tracking of the country’s progress in improving child health. However, the study is not without limitations. Though the GBD follows a well-established systematic and rigorous approach, the validity of the estimates is as good as the quality of input data, often extracted from different relevant sources. Due to the lack of vital registration systems in Ethiopia, the 2019 GBD study results for the country were primarily based on data that are not well designed and represent subnational realities. Moreover, it was also likely that regions with predominant pastoralist communities could have been underrepresented due to limited primary studies contributing to the estimates in these regions, partly due to no HDSS centres and difficult nature of data collections during DHS surveys and census enumerations. Hence, results should be considered with precautions. For example, in most datasets, those regions with smaller population sizes are poorly represented, which might have led to the underestimation of U5MR in these regions.

In addition, there is a possibility of misclassification of causes of death and risk factors. Neonatal and early childhood deaths may have coverage issues, particularly in rural areas and pastoralist regions. Despite such shortcomings in the estimates, this study provides a comprehensive assessment of mortality rates for national and its administrative regions, which will be helpful for monitoring the progress toward SDG targets. In the past few years, the country’s political situation has been volatile, involving conflict and huge population displacement, which might also affect the ability to identify and report deaths and their causes.

## Conclusions

Despite significant reductions in under-5, infant, and neonatal mortality over the past three decades, the rates remain unacceptably high. There were considerable regional disparities in the under-5, infant, and neonatal mortality rates in Ethiopia. The regions with predominant pastoralist communities bore a higher burden and lower reduction rates between 1990 and 2019. Addis Ababa, Dire Dawa, and Harari are on track to achieve the SDG and the National HSTP II targets in reducing under-5 mortality. A focused programme on neonates and younger children, such as access to essential health services, enhancing routine community health programmes and expanding critical neonatal health services should be devised and strengthened. Our study also highlights the urgent need for primary data collection, particularly in regions with predominantly pastoralist community, to improve the accuracy of regional under-5, infant, and neonatal mortality estimates.

## Supporting information

S1 Table Lists of causes of level 3 causes of death for under 5 children in GBD Study 2019*. *Presented alphabetically.
(DOCX)

S2 Table The rate of under-5, infant, and neonatal deaths by sex in 1990, 2000, 2015, 2019 in Ethiopia and sub-national regions death rate per 1000 livebirth (95% UI). ROC- Rate of change. UI—Uncertainty Interval. SNNPR-Southern Nations Nationality and People Region. *SNNPR comprises Sidama Region and Southwest Ethiopia Peoples’ Region.
(DOCX)

S3 Table Percentage of under-5 deaths attributable to each risk factors in Ethiopia and its regional states, 2019. *All risk factors column does not reflect the addition of groups of risk factors.
(DOCX)

S4 Table Percentage of infant deaths attributable to risk factors in Ethiopia and its regional states, 2019. *All risk factors column does not reflect the addition of groups of risk factors.
(DOCX)

S5 Table Percentage of neonatal deaths attributable to risk factors in Ethiopia and its regional states, 2019. *All risk factors column does not reflect the addition of groups of risk factors.
(DOCX)

S1 Fig Level three leading causes of infant mortality in Ethiopia in 1990 and 2019. *Neonatal disorders include preterm births, neonatal encephalopathy due to birth asphyxia and trauma, neonatal sepsis and other neonatal infectious, hemolytic diseases and other neonatal jaundice, and other neonatal disorders. STI- Sexual Transmitted Infections. HIV–Human Immunodeficiency virus. AIDS—Acquired Immunodeficiency Diseases. UI- Uncertainty interval.
(DOCX)

S2 Fig Level three leading causes of neonatal mortality in Ethiopia in 1990 and 2019. *Neonatal disorders include preterm births, neonatal encephalopathy due to birth asphyxia and trauma, neonatal sepsis and other neonatal infectious, hemolytic diseases and other neonatal jaundice, and other neonatal disorders. STI- Sexual Transmitted Infections. HIV–Human Immunodeficiency virus. AIDS—Acquired Immunodeficiency Diseases. UI- Uncertainty interval.
(DOCX)

S1 File GBD 2019 Ethiopia Child Mortality Collaborators. (DOCX)

S2 File GBD 2019 Ethiopia Child Mortality Collaborators authors’ contributions. (DOC)

S3 File GBD 2019 Ethiopia Child Mortality Collaborators list for PubMed indexing. (DOCX)
