# Supplementary material for: National and subnational burden of under-5, infant, and neonatal mortality in Ethiopia, 1990–2019: Findings from the Global Burden of Disease Study 2019
Source: PLOS Glob Public Health. 2023 Jun 21;3(6):e0001471. doi: 10.1371/journal.pgph.0001471 (PMC10284418; doi:10.1371/journal.pgph.0001471)
Supplement: S1 Table — *Presented alphabetically. (DOCX) [file pgph.0001471.s001.docx]

**S1 Table . Lists of causes of level 3 causes of death for under 5 children in GBD Study 2019***

| Acute glomerulonephritis | Leishmaniasis |
| --- | --- |
| Acute hepatitis | Leukemia |
| Adverse effects of medical treatment | Liver cancer |
| African trypanosomiasis | Lower respiratory infections |
| Animal contact | Malaria |
| Appendicitis | Malignant skin melanoma |
| Asthma | Measles |
| Bacterial skin diseases | Meningitis |
| Brain and central nervous system cancer | Motor neuron disease |
| Cardiomyopathy and myocarditis | Neonatal disorders |
| Chagas disease | Non-Hodgkin lymphoma |
| Chronic kidney disease | Other cardiovascular and circulatory diseases |
| Chronic obstructive pulmonary disease | Other chronic respiratory diseases |
| Cirrhosis and other chronic liver diseases | Other digestive diseases |
| Conflict and terrorism | Other intestinal infectious diseases |
| Congenital birth defects | Other malignant neoplasms |
| Cystic echinococcosis | Other neglected tropical diseases |
| Cysticercosis | Other neoplasms |
| Decubitus ulcer | Other neurological disorders |
| Dengue | Other nutritional deficiencies |
| Diabetes mellitus | Other skin and subcutaneous diseases |
| Diarrheal diseases | Other transport injuries |
| Diphtheria | Other unintentional injuries |
| Drowning | Other unspecified infectious diseases |
| Drug use disorders | Otitis media |
| Ebola | Pancreatitis |
| Encephalitis | Paralytic ileus and intestinal obstruction |
| Endocarditis | Poisonings |
| Endocrine, metabolic, blood, and immune disorders | Protein-energy malnutrition |
| Environmental heat and cold exposure | Rabies |
| Executions and police conflict | Rheumatic heart disease |
| Exposure to forces of nature | Road injuries |
| Exposure to mechanical forces | Schistosomiasis |
| Falls | Sexually transmitted infections excluding HIV |
| Fire, heat, and hot substances | Stroke |
| Foreign body | Sudden infant death syndrome |
| Gallbladder and biliary diseases | Testicular cancer |
| Hemoglobinopathies and hemolytic anemias | Tetanus |
| HIV/AIDS | Tuberculosis |
| Hodgkin lymphoma | Typhoid and paratyphoid |
| Idiopathic epilepsy | Upper digestive system diseases |
| Inflammatory bowel disease | Upper respiratory infections |
| Inguinal, femoral, and abdominal hernia | Urinary diseases and male infertility |
| Interpersonal violence | Varicella and herpes zoster |
| Interstitial lung disease and pulmonary sarcoidosis | Vascular intestinal disorders |
| Intestinal nematode infections | Whooping cough |
| Invasive Non-typhoidal Salmonella (iNTS) | Yellow fever |
| Kidney cancer | Zika virus |

**Presented alphabetically*
