# Supplementary material for: National and subnational burden of under-5, infant, and neonatal mortality in Ethiopia, 1990–2019: Findings from the Global Burden of Disease Study 2019
Source: PLOS Glob Public Health. 2023 Jun 21;3(6):e0001471. doi: 10.1371/journal.pgph.0001471 (PMC10284418; doi:10.1371/journal.pgph.0001471)
Supplement: S2 Table — ROC- Rate of change. UI—Uncertainty Interval. SNNPR-Southern Nations Nationality and People Region. *SNNPR comprises Sidama Region and Southwest Ethiopia Peoples’ Region. (DOCX) [file pgph.0001471.s002.docx]

**S2 Table. The rate of under-5, infant, and neonatal deaths by sex in 1990, 2000, 2015, 2019 in Ethiopia and sub-national regions death rate per 1000 livebirth (95% UI)**

| Country/ region | 1990 | | 2000 | | 2015 | | 2019 | |
| --- | --- | --- | --- | --- | --- | --- | --- | --- |
|  | **Male** | **Female** | **Male** | **Female** | **Male** | **Female** | **Male** | **Female** |
| **U5MR per 1,000 live births** | | | | | | | | |
| Ethiopia | 208.2 (191.6, 225.5) | 181 (169.3, 193.6) | 155.8 (144.1, 169.3) | 133.8 (123.5, 145.2) | 72.6 (62, 84.2) | 59.4 (52.5, 66.9) | 57.7 (48.4, 69.7) | 46.8 (40.7, 54.6) |
| Tigray | 188.2 (172.3, 205) | 172.6 (158.4, 188.6) | 129.1 (116.9, 141.1) | 116.7 (105.8, 128.7) | 48.9 (41.2, 57.2) | 41.8 (36.3, 47.6) | 35.9 (29.9, 43.7) | 30.2 (26.2, 35.4) |
| Afar | 185.2 (169.3, 202.1) | 170 (155.3, 186.2) | 138.1 (125.1, 150.7) | 125.5 (115.0, 136.5) | 58.5 (49.2, 69.3) | 50.5 (43.5, 57.6) | 46.2 (38.2, 56.8) | 39.4 (33.9, 46.6) |
| Amhara | 211.7 (196.7, 228.7) | 197.3 (183.2, 212.9) | 160.7 (147.5, 175.5) | 146.6 (133.9, 160.5) | 76.5 (62.1, 92.5) | 66.7 (55.7, 79.6) | 60.6 (50.8, 73.3) | 52.4 (45.7, 60.8) |
| Oromia | 203.8 (187.6, 221.5) | 187.5 (172.8, 204.2) | 149.5 (137.4, 163.7) | 135.8 (125.4, 147.5) | 70.5 (60.8, 81.2) | 61.3 (54.5, 69.1) | 55.7 (46.6, 67.5) | 47.9 (41.8, 55.8) |
| Somali | 148.3 (136.1, 161) | 134.7 (123.6, 146.6) | 126.2 (114.9, 138.1) | 114.4 (103.6, 126.1) | 79.4 (67.3, 92.7) | 69.4 (60.5, 78.6) | 67.2 (56.3, 81.5) | 58.3 (50.8, 67.9) |
| Benshangul-Gumuz | 243.1 (221.1, 268.6) | 225.6 (206.1, 246.9) | 199.5 (172.4, 228.9) | 183.5 (161.5, 207.3) | 96.7 (80.2, 114.1) | 85.1 (72.6, 98.4) | 77.8 (65.3, 94) | 67.9 (59.3, 78.7) |
| SNNPR | 221.5 (203.4, 240.1) | 204.5 (187.8, 223.2) | 167.3 (152.9, 185.6) | 152.7 (137.9, 173) | 72.8 (62.2, 84.7) | 63.4 (55.6, 71.9) | 40.3 (33.6, 49) | 34.1 (29.6, 39.9) |
| Gambella | 218.2 (200.4, 237.1) | 201.2 (184.3, 218.8) | 151.9 (139.3, 166.6) | 138.3 (125.2, 151.8) | 55.5 (47.3, 64.8) | 47.8 (41.6, 54.5) | 57.3 (48, 69.3) | 49.4 (43.1, 57.4) |
| Harari | 201.7 (183.7, 220.7) | 185.5 (170.2, 202.2) | 128.5 (110.5, 148.4) | 116.4 (100.2, 134.6) | 63 (53.7, 73.6) | 54.6 (47.7, 62) | 51.3 (42.9, 62.2) | 44 (38.3, 51.3) |
| Dire Dawa | 202.9 (184.8, 222.4) | 186.8 (171.2, 203.7) | 135.9 (117.8, 157.6) | 123.4 (106.9, 142.5) | 65 (55.3, 76) | 56.3 (49.1, 64.2) | 51.2 (42.8, 62.2) | 43.9 (38.2, 51.3) |
| Addis Ababa | 99.3 (88.9, 110.7) | 92.1 (84, 100.8) | 74.2 (62.2, 87.7) | 67.1 (57.6, 77.4) | 21.4 (17.3, 25.9) | 17.6 (14.6, 20.9) | 16.1 (13.3, 19.8) | 13.3 (11.4, 15.8) |
| **IMR per 1,000 live births** | | | | | | | | |
| Ethiopia | 135.2 (121.4, 149.9) | 108.9 (100.8, 117.4) | 105.4 (93.9, 118.6) | 82.2 (76.0, 89.4) | 55.6 (46.8, 65.4) | 42 (36.6, 48.2) | 45.0 (37.6, 54.7) | 34.1 (29.6, 39.9) |
| Tigray | 130.8 (118.4, 144.4) | 112.8 (101.9, 124.6) | 94.5 (85, 104.2) | 79.9 (71.7, 89.1) | 40.2 (33.7, 47.4) | 33.1 (28.6, 37.9) | 29.8 (24.7, 36.5) | 24.4 (21, 28.7) |
| Afar | 128.6 (116.4, 142.2) | 110.8 (100.2, 122.7) | 99.8 (89.3, 110.5) | 84.5 (76.8, 92.5) | 47.2 (39.4, 56.3) | 38.8 (33.3, 44.6) | 37.8 (31, 46.8) | 31 (26.5, 36.9) |
| Amhara | 143.2 (131, 156.7) | 125.1 (114.6, 137.2) | 114.4 (104.3, 126.1) | 97.4 (87.7, 107.7) | 60.5 (48.6, 73.7) | 49.9 (41.3, 60) | 48.9 (40.7, 59.7) | 40.2 (34.9, 47.1) |
| Oromia | 140.2 (127.2, 154.1) | 121.2 (109.9, 134) | 107.7 (97.6, 119.1) | 91.4 (84.1, 99.9) | 56.3 (48.1, 65.6) | 46.4 (41, 52.6) | 45.3 (37.6, 55.4) | 37.3 (32.3, 43.7) |
| Somali | 105.8 (96.4, 116) | 90.3 (82.1, 99.5) | 91.9 (82.9, 101.5) | 77.7 (69.7, 86.7) | 62.1 (52, 73.2) | 51.1 (44.2, 58.3) | 53.3 (44.2, 65.4) | 43.9 (37.9, 51.5) |
| Benshangul-Gumuz | 162.5 (144.3, 183.2) | 141.9 (127.8, 157) | 138.2 (116.6, 162.4) | 118.5 (102.8, 136.3) | 74.6 (61.3, 89.2) | 61.6 (52.1, 71.8) | 61.3 (51, 75.1) | 50.5 (43.8, 59.2) |
| SNNPR | 150.5 (136.3, 165.7) | 130.7 (118.5, 144.6) | 118.8 (108.1, 131) | 101.3 (91.4, 113.5) | 58 (49.2, 68) | 47.8 (41.6, 54.5) | 33.5 (27.8, 41.1) | 27.5 (23.7, 32.4) |
| Gambella | 149.1 (134.8, 164.1) | 129.4 (116.8, 143.2) | 109.6 (99.5, 121.3) | 93.1 (83.5, 103.4) | 45.5 (38.5, 53.5) | 37.5 (32.5, 43) | 46.5 (38.7, 56.8) | 38.3 (33.2, 44.8) |
| Harari | 141.3 (126.9, 157.6) | 122.2 (110.4, 134.8) | 95.7 (81.1, 112.1) | 81.1 (68.8, 94.9) | 51.5 (43.5, 60.7) | 42.5 (36.9, 48.6) | 42.5 (35.3, 52) | 35 (30.3, 41.1) |
| Dire Dawa | 141.2 (126.4, 157.9) | 122.3 (110.4, 135.1) | 100.2 (85.6, 117.6) | 85 (72.5, 99.8) | 52.9 (44.7, 62.5) | 43.7 (37.9, 50) | 42.3 (35.1, 51.9) | 34.9 (30.1, 41) |
| Addis Ababa | 72.7 (64.2, 82.3) | 62.8 (56.7, 69.7) | 57.5 (47.3, 69) | 48.8 (41.1, 57.2) | 17.9 (14.4, 21.7) | 14.5 (12, 17.2) | 13.3 (11, 16.4) | 10.9 (9.3, 12.9) |
| **NMR per 1,000 live births** | | | | | | | | |
| Ethiopia | 64.3 (58.3, 70.6) | 45.7 (42.5, 49) | 56.8 (51.1, 63.5) | 39.4 (36.5, 42.6) | 36.8 (31.1, 43.1) | 26.1 (22.8, 29.8) | 31.0 (26.0, 37.4) | 21.9 (19, 25.5) |
| Tigray | 48.1 (43.8, 52.7) | 39.5 (35.8, 43.4) | 38.9 (35.1, 42.6) | 30.9 (27.9, 34.3) | 23.5 (19.7, 27.6) | 18.3 (15.8, 20.9) | 18.6 (15.5, 22.8) | 14.5 (12.5, 17) |
| Afar | 47.6 (43.3, 52.3) | 39 (35.4, 43) | 40.7 (36.6, 44.9) | 32.5 (29.7, 35.3) | 25.9 (21.7, 30.9) | 20.1 (17.3, 23.1) | 22.2 (18.3, 27.5) | 17.3 (14.8, 20.5) |
| Amhara | 51.4 (47.3, 55.9) | 42.9 (39.4, 46.8) | 44.4 (40.8, 48.7) | 35.6 (32.3, 39.3) | 30.4 (24.5, 36.9) | 23.6 (19.6, 28.3) | 26.4 (22, 32.1) | 20.4 (17.8, 23.9) |
| Oromia | 50.7 (46.3, 55.3) | 41.8 (38, 45.9) | 43.2 (39.3, 47.6) | 34.5 (31.9, 37.4) | 29.2 (25, 33.9) | 22.7 (20, 25.6) | 25.2 (21, 30.7) | 19.5 (16.9, 22.9) |
| Somali | 41.2 (37.7, 44.9) | 33.3 (30.5, 36.5) | 38.6 (35, 42.4) | 30.7 (27.7, 34.2) | 30.9 (26, 36.4) | 24 (20.8, 27.4) | 28.0 (23.3, 34.2) | 21.7 (18.8, 25.4) |
| Benshangul-Gumuz | 56.9 (50.8, 63.9) | 47.5 (43.2, 52) | 51.8 (44.2, 60.4) | 42 (36.8, 47.8) | 35.1 (29, 41.7) | 27.3 (23.1, 31.7) | 30.6 (25.5, 37.4) | 23.7 (20.6, 27.8) |
| SNNPR | 53.9 (49.1, 59.1) | 44.6 (40.6, 49.1) | 46.7 (42.7, 51.1) | 37.6 (34, 41.9) | 29.8 (25.4, 34.9) | 23.1 (20.2, 26.4) | 20.1 (16.7, 24.6) | 15.6 (13.5, 18.3) |
| Gambella | 53.2 (48.4, 58.2) | 44 (40, 48.4) | 42.9 (39.1, 47.2) | 34.3 (30.9, 37.9) | 25 (21.3, 29.3) | 19.5 (16.9, 22.3) | 25.7 (21.5, 31.4) | 20 (17.3, 23.4) |
| Harari | 51.3 (46.2, 56.8) | 42.3 (38.4, 46.4) | 39.4 (33.7, 45.6) | 31.3 (26.8, 36.4) | 27.1 (23, 31.8) | 21 (18.3, 24) | 23.8 (19.8, 29.1) | 18.5 (16, 21.7) |
| Dire Dawa | 50.7 (45.7, 56.4) | 41.9 (37.9, 45.9) | 40.3 (34.7, 47.2) | 32.1 (27.6, 37.4) | 27.7 (23.4, 32.6) | 21.5 (18.7, 24.6) | 23.7 (19.7, 29) | 18.4 (15.9, 21.6) |
| Addis Ababa | 31.2 (27.6, 35.2) | 25.5 (23.1, 28.2) | 26.9 (22.2, 32.1) | 21.1 (17.9, 24.7) | 11.3 (9.2, 13.7) | 8.8 (7.3, 10.4) | 8.5 (7.0, 10.5) | 6.7 (5.7, 8) |

U5MR – Under 5 mortality Rate, IMR- Infant Mortality Rate, NMR- Neonatal Mortality Rate, UI – Uncertainty Interval. AROC – Annual Rate of Change, SNNPR**-**Southern Nations, Nationality, and People’s Region
