# Supplementary material for: National and subnational burden of under-5, infant, and neonatal mortality in Ethiopia, 1990–2019: Findings from the Global Burden of Disease Study 2019
Source: PLOS Glob Public Health. 2023 Jun 21;3(6):e0001471. doi: 10.1371/journal.pgph.0001471 (PMC10284418; doi:10.1371/journal.pgph.0001471)
Supplement: S3 Table — *All risk factors column does not reflect the addition of groups of risk factors. (DOCX) [file pgph.0001471.s003.docx]

S3 Table. Percentage of under-5 deaths attributable to each risk factors in Ethiopia and its regional states, 2019

| **Country/ region** | **Child and maternal malnutrition** | | | **Unsafe water, sanitation, and handwashing (WaSH)** | | | | **Air pollution** | | |  |
| --- | --- | --- | --- | --- | --- | --- | --- | --- | --- | --- | --- |
|  | **Total** | **Low birth weight and short gestation** | **Child growth failure** | **Total** | **Unsafe water** | **No access to handwashing facility** | **Unsafe sanitation** | **Total** | **Ambient particulate matter pollution** | **Household air pollution from solid fuels** | **All risk factors*** |
| Ethiopia | 57.2 (51.3-62.8) | 36.8 (34.8-38.7) | 20 (15.2-24.8) | 15.2 (10.9-21.3) | 11.3 (7.6-17.1) | 6.8 (4.6-9.2) | 8.8 (6.2-12.9) | 15.9 (13.8-18) | 1.5 (0.6-2.9) | 14.5 (11.9-16.8) | 61.6 (55.1-67.8) |
| Tigray | 57.2 (49.3-63.5) | 41.4 (38.2-44.5) | 15.5 (9.4-20.2) | 13.4 (8-19) | 9.8 (5.2-15) | 6 (3.6-8.4) | 7.5 (4.2-11.2) | 17.1 (13.5-20.8) | 2.3 (0.9-4.7) | 14.7 (10.8-18.7) | 61.4 (52.8-68.1) |
| Afar | 59.2 (50.2-66.4) | 39.3 (36.9-41.6) | 19.5 (11-25.8) | 13.8 (8.1-19.6) | 10 (5.2-15.4) | 6.4 (3.8-9.1) | 7.9 (4.2-11.8) | 17 (13.7-20.5) | 1.4 (0.4-3.2) | 15.7 (12.1-19.3) | 63 (53.3-70.5) |
| Amhara | 52.3 (44.5-61.7) | 34.3 (31.5-36.6) | 17.7 (11.1-26.1) | 14.5 (7.9-26.1) | 11.2 (5.5-22.6) | 6.2 (3.5-10.1) | 8.8 (4.5-17.2) | 14.1 (10.9-17) | 1.4 (0.5-3) | 12.7 (9.3-15.7) | 56.4 (48-67) |
| Oromia | 61 (54.3-67) | 39 (36.9-41) | 21.5 (15.8-26.5) | 16.7 (11.7-22.8) | 12.5 (8.1-18.1) | 7.4 (5-10) | 9.7 (6.5-13.8) | 16.9 (13.7-19.9) | 1.5 (0.6-3) | 15.3 (11.9-18.6) | 65.4 (57.8-72.1) |
| Somali | 59.2 (51.3-65.6) | 35.2 (33.1-37.1) | 23.5 (16.1-29) | 12.9 (8.1-18.6) | 8.6 (4.7-13.4) | 6.6 (4.1-9.4) | 6.6 (3.8-10.3) | 18.4 (15-22.3) | 0.8 (0.2-2.1) | 17.6 (14.1-21.5) | 62.9 (54.5-69.5) |
| Benishangul-Gumuz | 52.5 (44.2-59.8) | 31 (28.9-33.1) | 21.1 (13.8-27.2) | 12.3 (7.2-19.6) | 8.1 (4-14.3) | 6.4 (3.8-9.7) | 6.3 (3.2-11.4) | 15.9 (12.3-19.6) | 1.6 (0.6-3.4) | 14.3 (10.3-18.1) | 57.6 (48.2-65.8) |
| SNNPR | 55 (46.8-61.5) | 35.3 (32.8-37.6) | 19.3 (12.2-25) | 15 (9.1-21.8) | 11.2 (6.2-17.9) | 6.7 (4.1-9.4) | 8.7 (5-13.3) | 15 (11.8-18.2) | 1.4 (0.5-2.8) | 13.5 (10.3-16.9) | 59.7 (50.6-67.1) |
| Gambella | 47.8 (39.4-55.1) | 38 (33.2-41.8) | 9.6 (4.4-14.9) | 7.7 (3.6-12.4) | 5.2 (2.2-9.4) | 3.7 (1.8-5.9) | 4.2 (1.8-7.4) | 14.7 (10.7-18.4) | 2.4 (0.9-4.7) | 12.3 (8.6-16) | 50.9 (41.9-59.2) |
| Harari | 46.4 (38.3-54.1) | 34.1 (30.3-37) | 12.1 (6.1-18.6) | 9.2 (4.5-16.1) | 5.8 (2.3-11.4) | 4.3 (2.2-7.1) | 5.1 (2.2-9.5) | 11.5 (7.9-15.4) | 2.9 (1.3-5.1) | 8.6 (5.3-12.3) | 50.2 (40.9-59.3) |
| Dire Dawa | 47.6 (38.8-56) | 35.9 (32.2-39) | 11.4 (5.2-18.2) | 8.3 (3.6-15.4) | 5.6 (2-11.5) | 4.1 (1.9-7) | 4.3 (1.6-8.8) | 12.4 (8.9-16.3) | 3.3 (1.6-5.9) | 9 (5.7-12.8) | 50.9 (41.2-60.2) |
| Addis Ababa | 47.1 (38.4-54) | 38.6 (31.7-43.9) | 8.2 (5-11.5) | 6.6 (4.1-9.4) | 2.9 (1.2-5.3) | 3.1 (2-4.5) | 3.3 (1.9-5) | 8.6 (4.6-12.8) | 5.7 (2.9-9.1) | 2.9 (1.2-5.3) | 49.6 (40.4-57.1) |

*All risk factors column does not reflect the addition of groups of risk factors
