# Supplementary material for: National and subnational burden of under-5, infant, and neonatal mortality in Ethiopia, 1990–2019: Findings from the Global Burden of Disease Study 2019
Source: PLOS Glob Public Health. 2023 Jun 21;3(6):e0001471. doi: 10.1371/journal.pgph.0001471 (PMC10284418; doi:10.1371/journal.pgph.0001471)
Supplement: S4 Table — *All risk factors column does not reflect the addition of groups of risk factors. (DOCX) [file pgph.0001471.s004.docx]

**S4 Table. Percentage of infant deaths attributable to risk factors in Ethiopia and its regional states, 2019**

| **Country/ region** | **Child and maternal malnutrition†** | | | **Unsafe water, sanitation, and handwashing (WaSH)** | | | | **Air pollution** | | | **All risk factors*** |
| --- | --- | --- | --- | --- | --- | --- | --- | --- | --- | --- | --- |
|  | **Total** | **Low birth weight and short gestation** | **Child growth failure** | **Total** | **Unsafe water** | **No access to handwashing facility** | **Unsafe sanitation** | **Total** | **Ambient particulate matter pollution** | **Household air pollution from solid fuels** |  |
| Ethiopia | 63.6 (59-68.1) | 49.6 (46.9-52) | 13.6 (10.7-16.5) | 11.5 (8.5-16.2) | 8.2 (5.5-12.4) | 5.5 (3.8-7.4) | 6.4 (4.5-9.5) | 19.3 (16.8-21.5) | 1.8 (0.7-3.5) | 17.5 (14.6-20.2) | 68.0 (62.9-72.7) |
| Tigray | 63.9 (57.2-69.8) | 53 (49-56.9) | 10.6 (6.9-14) | 10.4 (6.6-15.1) | 7.2 (3.9-11.7) | 5 (3.2-7) | 5.5 (3.1-8.6) | 20.5 (16.3-25) | 2.8 (1-5.6) | 17.7 (13.1-22.5) | 68.1 (60.9-74.1) |
| Afar | 65.2 (59-70.7) | 52.1 (48.9-55) | 12.8 (7.6-16.8) | 10.7 (6.8-15.5) | 7.3 (4.1-11.6) | 5.3 (3.4-7.5) | 5.8 (3.3-8.8) | 20.4 (16.8-24.4) | 1.6 (0.5-3.8) | 18.8 (14.9-23) | 69.1 (62.6-74.9) |
| Amhara | 58.6 (52.1-66.4) | 46.8 (42.9-49.9) | 11.6 (7.4-17.5) | 10.3 (5.5-19.5) | 7.5 (3.3-16.3) | 4.8 (2.6-7.9) | 5.9 (2.7-12.2) | 17.8 (13.6-21.6) | 1.7 (0.6-3.8) | 16.1 (11.9-20) | 63 (56-71.5) |
| Oromia | 67.5 (62.3-72.3) | 52.2 (49.3-54.9) | 14.9 (11.3-18) | 12.8 (8.8-17.7) | 9.2 (5.6-14.2) | 5.9 (4-8.1) | 7.2 (4.6-10.8) | 20.1 (16.1-23.8) | 1.8 (0.7-3.6) | 18.3 (14.1-22.3) | 71.7 (66-76.6) |
| Somali | 65.3 (59.6-70.5) | 49.1 (46.2-51.7) | 15.6 (11-19.2) | 10.3 (6.6-14.8) | 6.5 (3.4-10.5) | 5.6 (3.5-7.8) | 5 (2.6-7.9) | 22.1 (17.9-26.1) | 1 (0.3-2.4) | 21.1 (16.9-25.2) | 69.3 (63.4-74.6) |
| Benishangul-Gumuz | 59.4 (52.6-65.4) | 44.1 (41-46.8) | 15 (10.3-19.4) | 9.8 (5.7-15.8) | 6 (2.8-11.2) | 5.4 (3.1-8) | 4.7 (2.2-8.5) | 19 (14.7-23.3) | 1.9 (0.7-4.1) | 17.1 (12.6-21.5) | 64.4 (57.3-70.8) |
| SNNPR | 61.1 (54.6-66.4) | 47.4 (44.1-50.4) | 13.2 (8.7-17.2) | 11.5 (7.5-17.3) | 8.1 (4.7-13.4) | 5.5 (3.5-7.7) | 6.3 (3.8-10.1) | 18 (14.1-21.9) | 1.7 (0.6-3.3) | 16.3 (12.4-20.3) | 65.9 (58.8-71.8) |
| Gambella | 55.9 (47.9-63) | 48.6 (42.5-53.5) | 7 (3.4-10.6) | 6.4 (3.3-10.3) | 4 (1.7-7.4) | 3.3 (1.7-5.1) | 3.2 (1.4-5.9) | 17.9 (13-22.3) | 2.9 (1.1-5.7) | 15.1 (10.4-19.5) | 59.2 (50.6-66.5) |
| Harari | 55.1 (47.2-61.9) | 45.1 (40.1-48.9) | 9.7 (5-14.3) | 7.6 (3.9-12.8) | 4.4 (1.8-8.5) | 3.9 (2-6.1) | 3.9 (1.7-7.1) | 14.3 (9.9-18.8) | 3.6 (1.7-6.4) | 10.7 (6.6-15.2) | 58.9 (50.3-66.2) |
| Dire Dawa | 56.5 (48.9-64) | 47.5 (42.6-51.4) | 8.8 (4.4-13.6) | 7 (3.2-12.7) | 4.3 (1.5-9) | 3.6 (1.8-6.2) | 3.3 (1.2-7) | 15.5 (11.2-20) | 4.1 (1.9-7.3) | 11.3 (7.2-15.7) | 60.1 (51.8-68) |
| Addis Ababa | 56.6 (46.3-64.7) | 49.5 (40.7-56.4) | 6.8 (4.3-9.6) | 6 (3.9-8.8) | 2.5 (1-4.6) | 3 (1.9-4.2) | 2.8 (1.7-4.4) | 10.7 (5.6-16) | 7.1 (3.5-11.3) | 3.6 (1.5-6.7) | 58.8 (48.2-67.3) |

*All risk factors column does not reflect the addition of groups of risk factors
