# Supplementary material for: National and subnational burden of under-5, infant, and neonatal mortality in Ethiopia, 1990–2019: Findings from the Global Burden of Disease Study 2019
Source: PLOS Glob Public Health. 2023 Jun 21;3(6):e0001471. doi: 10.1371/journal.pgph.0001471 (PMC10284418; doi:10.1371/journal.pgph.0001471)
Supplement: S5 Table — *All risk factors column does not reflect the addition of groups of risk factors. (DOCX) [file pgph.0001471.s005.docx]

**S5 Table. Percentage of neonatal deaths attributable to risk factors in Ethiopia and its regional states, 2019**

| **Country/ region** | **Low birth weight and short gestation** | **Unsafe water, sanitation, and handwashing** | | | **Air pollution** | | | | **All risk factors*** |
| --- | --- | --- | --- | --- | --- | --- | --- | --- | --- |
|  |  | **Total** | **Unsafe water** | **No access to handwashing facility** | **Unsafe sanitation** | **Total** | **Ambient particulate matter pollution** | **Household air pollution from solid fuels** |  |
| Ethiopia | 71.1 (67.2-74.6) | 3.8 (2.6-5.3) | 1.6 (1-2.5) | 2.2 (1.4-3.2) | 1.6 (1-2.5) | 23.3 (20.4-26) | 2.1 (0.8-4.1) | 21.3 (17.9-24.4) | 75.8 (71.7-79.4) |
| Tigray | 70.4 (65.1-75.5) | 4 (2.6-6.1) | 1.6 (0.9-2.9) | 2.4 (1.4-3.6) | 1.6 (0.9-2.9) | 24.4 (19.1-29.7) | 3.3 (1.2-6.6) | 21.1 (15.6-26.6) | 74.9 (69.4-80) |
| Afar | 71.9 (67.5-75.9) | 4.2 (2.9-6) | 1.8 (1.1-2.7) | 2.6 (1.6-3.7) | 1.8 (1.1-2.7) | 24.3 (19.8-28.7) | 1.9 (0.6-4.5) | 22.4 (18-27.2) | 76.4 (72-80.6) |
| Amhara | 68 (62.2-72.7) | 2.8 (1.4-5.5) | 1.1 (0.4-2.9) | 1.8 (0.9-3) | 1.1 (0.4-2.9) | 22.7 (17.3-27.5) | 2.2 (0.7-4.8) | 20.5 (15.2-25.4) | 72.9 (66.8-78.1) |
| Oromia | 74.4 (70.5-78.1) | 4.1 (2.4-6.4) | 1.9 (0.9-3.4) | 2.3 (1.4-3.5) | 1.9 (0.9-3.4) | 24.3 (19.3-29.1) | 2.1 (0.8-4.4) | 22.2 (17.1-27.1) | 78.7 (74.6-82.4) |
| Somali | 72.8 (68.6-76.7) | 3.9 (2.4-5.7) | 1.4 (0.8-2.5) | 2.6 (1.4-3.8) | 1.4 (0.8-2.5) | 26 (21-30.7) | 1.1 (0.3-2.9) | 24.9 (20.1-29.8) | 77.6 (73.3-81.6) |
| Benishangul-Gumuz | 67.2 (62.6-71.4) | 3.5 (2-5.8) | 1.3 (0.6-2.6) | 2.3 (1.2-3.6) | 1.3 (0.6-2.6) | 22.2 (16.6-27.3) | 2.1 (0.8-4.8) | 20.1 (14.6-25.2) | 73 (67.8-77.4) |
| Southern Nations, | 67.8 (63.2-71.9) | 3.9 (2.4-6.1) | 1.7 (0.9-3.1) | 2.3 (1.4-3.3) | 1.7 (0.9-3.1) | 21.4 (16.5-26.4) | 1.9 (0.7-3.9) | 19.5 (14.7-24.6) | 73.2 (68.3-77.6) |
| Gambella | 66.3 (57.9-73) | 2.7 (1.5-4.3) | 1 (0.5-2) | 1.8 (0.9-2.8) | 1 (0.5-2) | 22.2 (16.1-27.9) | 3.5 (1.4-6.9) | 18.7 (13-24.4) | 70.1 (61.2-77) |
| Harari | 64.3 (57.2-69.8) | 3 (1.7-5.3) | 1.1 (0.5-2.3) | 1.9 (1-3.3) | 1.1 (0.5-2.3) | 17.8 (11.7-23.9) | 4.4 (2.0-8.0) | 13.4 (8.1-18.9) | 69 (61.7-74.8) |
| Dire Dawa | 67.7 (60.9-73.3) | 2.7 (1.4-5.3) | 1 (0.4-2.3) | 1.8 (0.9-3) | 1 (0.4-2.3) | 19.5 (13.9-25.6) | 5.2 (2.3-9.4) | 14.4 (9-19.9) | 71.7 (64.7-77.8) |
| Addis Ababa | 65.4 (53.7-74.4) | 2.8 (1.7-4.1) | 1.1 (0.6-1.8) | 1.7 (1-2.5) | 1.1 (0.6-1.8) | 13.3 (6.5-20) | 8.8 (4-14.2) | 4.5 (1.8-8.4) | 67.5 (55.4-76.7) |

*All risk factors column does not reflect the addition of groups of risk factors.
