# Supplementary material for: National and subnational burden of under-5, infant, and neonatal mortality in Ethiopia, 1990–2019: Findings from the Global Burden of Disease Study 2019
Source: PLOS Glob Public Health. 2023 Jun 21;3(6):e0001471. doi: 10.1371/journal.pgph.0001471 (PMC10284418; doi:10.1371/journal.pgph.0001471)
Supplement: S2 Fig — *Neonatal disorders include preterm births, neonatal encephalopathy due to birth asphyxia and trauma, neonatal sepsis and other neonatal infectious, hemolytic diseases and other neonatal jaundice, and other neonatal disorders. STI- Sexual Transmitted Infections. HIV–Human Immunodeficiency virus. AIDS—Acquired Immunodeficiency Diseases. UI- Uncertainty interval. (DOCX) [file pgph.0001471.s007.docx]

S2 Fig. Level three leading causes of neonatal mortality in Ethiopia in 1990 and 2019

*Neonatal disorders include preterm births, neonatal encephalopathy due to birth asphyxia and trauma, neonatal sepsis and other neonatal infectious, hemolytic diseases and other neonatal jaundice, and other neonatal disorders. STI- Sexual Transmitted Infections. HIV– Human Immunodeficiency virus. AIDS - Acquired Immunodeficiency Diseases. UI- Uncertainty interval.
