# Supplementary material for: National and subnational burden of under-5, infant, and neonatal mortality in Ethiopia, 1990–2019: Findings from the Global Burden of Disease Study 2019
Source: PLOS Glob Public Health. 2023 Jun 21;3(6):e0001471. doi: 10.1371/journal.pgph.0001471 (PMC10284418; doi:10.1371/journal.pgph.0001471)
Supplement: S1 File — (DOCX) [file pgph.0001471.s008.docx]

**S1 File. GBD 2019 Ethiopia Child Mortality Collaborators**

Gizachew A Tessema,^1,2*^ Tezera Moshago Berheto,^3*^ Berihun Assefa Dachew,^1,4^ Yohannes Adama Melaku,^5,6^ Zohra S Lassi,^7,8^ Kedir Hussein Abegaz,^9,10^ Tadesse M Abegaz,^11,12^ Kidist Adamu,^13^ Mohammed Hussien Adem,^14^ Muktar Beshir Ahmed,^15,16^ Gizachew Taddesse Akalu,^17,18^ Mastewal Belayneh Aklil,^19^ Addis Aklilu,^20^ Abayneh Tadesse Alamer,^21^ Dejene Tsegaye Alem,^22^ Addisu Alehegn Alemu,^23^ Musa Mohammed Ali,^24^ Hiwot Amare,^25^ Daniel Atlaw,^26^ Atalel Fentahun Awedew,^27^ Nefsu Awoke,^28^ Tewachew Awoke,^29^ Tegegn Mulatu Ayana,^30^ Solomon Shitu Ayen,^31^ Niguss Cherie Bekele,^32^ Melaku Ashagrie Belete,^33^ Alemshet Yirga Berhie,^34^ Belay Boda Abule Bodicha,^35^ Chuchu Churko,^36^ Abel Fekadu Dadi,^37,38^ Wakgari Binu Daga,^39^ Natanim Degefu,^40^ Tadesse Mamo Dejene,^41^ Getnet Makasha Demeke,^42,43^ Meseret Derbew Molla,^44^ Msganaw Derese,^45^ Kebede Deribe,^46,47^ Amare Deribew,^48,49^ Abebaw Alemayehu Desta,^50^ Aklilu Endalamaw,^51,52^ Getnet Gedif Engida,^53^ Daniel Berhanie Enyew,^54^ Tahir Eyayu,^55^ Addis Eyeberu,^56^ Demissie Assegu Fenta,^57^ Zinabu Fentaw,^58^ Tomas Y Ferede,^59^Daniel Baza Gargamo,^60^ Mesfin Gebrehiwot,^61^ Amanuel Tesfay Gebremedhin,^62,1^ Teferi Gebru Gebremeskel,^63,64^ Mathewos Alemu Gebremichael,^65^ Yalemzewod Assefa Gelaw,^66,1,^ Getachew Tilahun Gessese,^67^ Lemma Getacher,^68^ Melaku Getachew,^69^ Motuma Erena Getachew,^70,71^ Tamirat Getachew,^72^ Alene Geteneh,^73^ Fentabil Getnet,^74,75^ Abraham Tamirat Gizaw,^76^ Dessalegn Geleta Gobena,^77^ Temesgen Worku Gudayu,^78^ Abdiwahab Hashi,^79^ Simon I Hay,^80,81^ Demisu Zenbaba Heyi,^82^ Foziya Mohammed Hussien,^32^ Alelign Tasew Jema,^83^  Bedru Jemal,^84^ Girum Gebremeskel Kanno,^85^ Bekalu Getnet Kassa,^86^ Getahun Molla Kassa,^87^ Adera Debella Kebede,^56^ Worku Misganaw Kebede,^88^ Getiye Dejenu Kibret,^53^ Tebabere Moltot Kitaw,^89^ Mohammed Abdurke Kure,^90^ Galana Ayana Mamo,^91^ Maru Mekie,^86^ Bedasa Taye Merga,^92^ Belsity Temesgen Meselu,^23^ Gedefaye Nibret Mihrtie,^86^ Alemu Basazin Mingude,^88^ Hussen Mohammed,^93^ Mensur Shafie Mohammed,^94,95^ Salahuddin Mohammed,^96,97^ Zewdie Mulissa,^98^ Getaneh Baye Mulu,^99^ Beemnet Tekabe Mulugeta,^100^ Christopher J L Murray,^80,81^ Henok Biresaw Netsere,^101102^ Misganu Teshoma Regasa,^103^ Biniyam Sahiledengle,^104^ Endalew Gemechu Sendo,^105^ Nigussie Tadesse Sharew,^89,106^ Bereket Beyene Shashamo,^107^ Migbar Mekonnen Sibhat,^108^ Yitagesu Sintayehu,^109^ Yared Tadesse,^110^ Kasahun Girma Tareke,^76^ Mengistie Kassahun Tariku,^111^ Belay Negash Tefera,^112^ Getaye Worku Tesema,^113^ Gebiyaw Wudie Tsegaye,^114^ Biruk Shalmeno Tusa,^115^ Gebresilasea Gendisha Ukke,^116^ Birhanu Wagaye,^117,118^ Mandaras Tariku Walde,^119^ Meklit Girma Woldmicheal,^120^ Tewodros Eshete Wonde,^52^ Ayenew Engida Yismaw,^121^ Yazachew Yismaw,^122,123^ Gavin Pereira,^1,124$^ Awoke Misganaw,^81,125$^ Yohannes Kinfu.^126,127$^

* Joint first authors

^$^Joint last authors

# Affiliations

^1^Curtin School of Population Health, Curtin University, Perth, WA, Australia; ^2^School of Public Health, University of Adelaide, Adelaide, SA, Australia; ^3^HIV and TB Research Directorate, Ethiopian Public Health Institute, Addis Ababa, Ethiopia; ^4^Department of Epidemiology, University of Gondar, Gondar, Ethiopia; ^5^Adelaide Institute of Sleep Health, Flinders University, Adelaide, SA, Australia; ^6^Department of Public Health Nutrition, Bahir Dar University, Bahir Dar, SA, Ethiopia; ^7^Robinson Research Institute, University of Adelaide, Adelaide, SA, Australia; ^8^Department of Pediatrics, Aga Khan University, Karachi, Pakistan; ^9^Department of Biostatistics, Near East University, Nicosia, Cyprus; ^10^Department of Biostatistics and Health Informatics, Madda Walabu University, Bale Robe, Ethiopia; ^11^Department of Clinical Pharmacy, University of Gondar, Gondar, Ethiopia; ^12^College of Pharmacy and Pharmaceutical Sciences, Florida A&M University, Tallahassee, Florida, United States of America; ^13^Department of Health System Management, Wollo University, Dessie, Ethiopia; ^14^Department of Health Systems Management and Health Economics, Bahir Dar University, Bahir Dar, Ethiopia; ^15^Department of Epidemiology, Jimma University, Jimma, Ethiopia; ^16^Australian Center for Precision Health, University of South Australia, Adelaide, SA, Australia; ^17^Microbiology, Immunology and Parasitology Department, St. Paul's Hospital Millennium Medical College, Addis Ababa, Ethiopia; ^18^Microbial, Cellular and Molecular Biology Department, Addis Ababa University, Addis Ababa, Ethiopia; ^19^Department of Clinical Midwifery, Ethiopian Academy of Medical Science, Gondar, Ethiopia; ^20^Department of Medical Laboratory Sciences, Arba Minch University, Arba Minch, Ethiopia; ^21^Department of Physiotherapy, Mekelle University, Mekelle, Ethiopia; ^22^Department of Nursing, Debre Markos University, Debremarkos, Ethiopia; ^23^Midwifery Department, Debre Markos University, Debre Markos, Ethiopia; ^24^School of Medical Laboratory, Module of Microbiology and Parasitology, Hawassa University, Hawassa, Ethiopia; ^25^Department of Internal Medicine, Jimma University, Jimma, Ethiopia; ^26^Department of Biomedical Science, Madda Walabu University, Bale Robe, Ethiopia; ^27^Department of Surgery, Addis Ababa University, Addis Ababa, Ethiopia; ^28^Department of Nursing, Wolaita Sodo University, Wolaita Sodo, Ethiopia; ^29^Department of Medical Laboratory Sciences, Bahir Dar University, Bahir Dar, Ethiopia; ^30^School of Nursing, Arba Minch University, Arba Minch, Ethiopia; ^31^Department of Midwifery, Wolkite University, wolkite, Ethiopia; ^32^Department of Public Health, Wollo University, Dessie, Ethiopia; ^33^Medical Laboratory Science, Wollo University, Dessie, Ethiopia; ^34^School of Health Science, Bahir Dar University, Bahir Dar, Ethiopia; ^35^Biomedical Sciences Department, Arba Minch University, Arba Minch, Ethiopia; ^36^School of Public Health, Arba Minch University, Arba Minch, Ethiopia; ^37^Institute of Public Health, University of Gondar, Gondar, Ethiopia; ^38^Discipline of Public Health, Flinders University, Adelaide, SA, Australia; ^39^School of Public Health, Wolaita Sodo University, Sodo, Ethiopia; ^40^College of Health and Medical sciences, Haramaya University, Harar, Ethiopia; ^41^Department of Public Health, Debre Berhan University, Debre Berhan city, Ethiopia; ^42^college of Health Sciences, Debre Berhan University, Debere Berhan, Ethiopia; ^43^Department of Public Health, University of South Africa, Tswane, South Africa; ^44^Department of Biochemistry, University of Gondar, Gondar, Ethiopia; ^45^Department of Nursing, Mizan-Tepi University, Mizan-Aman, Ethiopia; ^46^Wellcome Trust Brighton and Sussex Centre for Global Health Research, Brighton and Sussex Medical School, Brighton, UK; ^47^School of Public Health, Addis Ababa University, Addis Ababa, Ethiopia; ^48^Nutrition International Ethiopia, Addis Ababa, Ethiopia; ^49^ School of Public Health, St. Paul Hospital Millennium Medical College, Addis Ababa, Ethiopia; ^50^Department of Surgical Nursing, University of Gondar, Gondar, Ethiopia; ^51^Department of Pediatrics and Child Health Nursing, Bahir Dar University, Bahir Dar, Ethiopia; ^52^School of Public Health, The University of Queensland, Brisbane, QLD, Australia; ^53^Department of Public Health, Debre Markos University, Debre Markos, Ethiopia; ^54^Department of Health Informatics, Haramaya University, Harar, Ethiopia; ^55^Department of Medical Laboratory Sciences, Debre Tabor University, Debre Tabor, Ethiopia; ^56^School of Nursing and Midwifery, Haramaya University, Harar, Ethiopia; ^57^School of Medical Laboratory Science, Hawassa University, Hawassa, Ethiopia; ^58^Department of Epidemiology and Biostatistics, Wollo University, Dessie, Ethiopia; ^59^School of Nursing, Hawassa University, Hawassa, Ethiopia; ^60^Pediatrics and Neonatal Nursing, Wolaita Sodo University, Wolaita Sodo, Ethiopia; ^61^Department of Environmental Health, Wollo University, Dessie, Ethiopia; ^62^Department of Population and Family Health, Jimma University, Jimma, Ethiopia; ^63^Discipline of Population Health, Flinders University, Adelaide, SA, Australia; ^64^Department of Reproductive Health, Aksum University, Aksum, Ethiopia; ^65^Department of Epidemiology and Biostatistics, Arba Minch University, Arba Minch, Ethiopia; ^66^Telethon Kids Institute, Nedlands, Western Australia, Australia; ^67^National Data Management Center, Ethiopian Public Health Institute, Addis Ababa, Ethiopia; ^68^Department of Public Health, Debre Berhan University, Debre Berhan, Ethiopia; ^69^Department of Emergency and Critical Care Medicine, Haramaya University, Harar, Ethiopia; ^70^Department of Public Health, Wollega University, Nekemte, Ethiopia; ^71^Department of Public Health, Jimma University, Jimma, Ethiopia; ^72^School of Nursing and Midwifery, Haramaya University, Harar, Ethiopia; ^73^Medical Laboratory Science Department, Woldia University, Woldia, Ethiopia; ^74^National Data Management Center, Ethiopian Health and Nutrition Research Institute, Addis Ababa, Ethiopia; ^75^Department of Epidemiology, Jigjiga University, Jigjiga, Ethiopia; ^76^Department of Health, Behavior and Society, Jimma University, Jimma, Ethiopia; ^77^Public Health Emergency Management Center, Ethiopian Health and Nutrition Research Institute, Addis Ababa, Ethiopia; ^78^Department of Clinical Midwifery, University of Gondar, Gondar, Ethiopia; ^79^Department of Public Health, Jigjiga University, Jigjiga, Ethiopia; ^80^Institute for Health Metrics and Evaluation, University of Washington, Seattle, Washington, United States of America; ^81^Department of Health Metrics Sciences, School of Medicine, University of Washington, Seattle, Washington, United States of America; ^82^Department of Public Health, Madda Walabu University, Robe, Goba Town, Ethiopia; ^83^Department of Public Health, Madda Walabu University, Bale Goba, Ethiopia; ^84^Department of Anesthesiology, Dilla University, Dilla, Ethiopia; ^85^School of Public Health, Dilla University, Dilla, Ethiopia; ^86^Department of Midwifery, Debre Tabor University, Debre Tabor, Ethiopia; ^87^Department of Epidemiology and Biostatistics, University of Gondar, Gondar, Ethiopia; ^88^Department of Nursing, Debre Berhan University, Debre Berhan, Ethiopia; ^89^Midwifery Department, Debre Berhan University, Debre Berhan, Ethiopia; ^90^College of Health and Medical Sciences, Haramaya University, Harar, Ethiopia; ^91^Department of Epidemiology and Biostatistics, Haramaya University, Harar, Ethiopia; ^92^School of Public Health, Haramaya University, Harar, Ethiopia; ^93^Department of Public Health, Dire Dawa University, Dire Dawa, Ethiopia; ^94^Pharmacology Department, St. Paul's Hospital Millennium Medical College, Addis Ababa, Ethiopia; ^95^Department of Pharmacology and Clinical Pharmacy, Addis Ababa University, Addis Ababa, Ethiopia; ^96^Department of Pharmaceutical Sciences, Notre Dame of Maryland University, Baltimore, Maryland, United States of America; ^97^Department of Pharmacy, Mizan-Tepi University, Mizan, Ethiopia; ^98^Accelerating Support to Advanced Local Partners (ASAP) Project, IntraHealth International, Addis Ababa, Ethiopia; ^99^Department of Pediatrics and Child Health Nursing, Debre Berhan University, Debre Berhan, Ethiopia; ^100^Department of Health Informatics, Arba Minch University, Arba Minch, Ethiopia; ^101^School of Nursing, University of Gondar, Gondar, Ethiopia; ^102^College of Medicine and Health Sciences, Bahir Dar University, Gondar, Ethiopia; ^103^Department of Midwifery, Wollega University, Nekemte, Ethiopia; ^104^Department of Public Health, Madda Walabu University, Bale Robe, Ethiopia; ^105^School of Nursing and Midwifery, Addis Ababa University, Addis Ababa, Ethiopia; ^106^Interdisciplinary Center Psychopathology and Emotion Regulation (ICPE), University of Groningen, Groningen, Netherlands; ^107^Department of Nursing, Arba Minch University, Arbaminch, Ethiopia; ^108^Department of Pediatrics and Child Health Nursing, Dilla University, Dilla, Ethiopia; ^109^Department of Midwifery, Dire Dawa University, Dire Dawa, Ethiopia; ^110^Maternal, Child and Adolescent Health Lead Executive Office, Federal Ministry of Health, Ethiopia; ^111^Department of Epidemiology and Biostatistics, Debre Markos University, Debre Markos, Ethiopia; ^112^Department of Public Health and Health Policy, Haramaya University, Harar, Ethiopia; ^113^Department of Midwifery, Ethiopian Public Health Institute, Addis Ababa, Ethiopia; ^114^College of Medicine and Health Sciences, Bahir Dar University, Bahir Dar, Ethiopia; ^115^Department of Epidemiology and Biostatistics, Haramaya University, Haramaya, Ethiopia; ^116^Department of Midwifery, Arba Minch University, Arba Minch, Ethiopia; ^117^Department of Public Health Nutrition, Wollo University, Dessie, Ethiopia; ^118^Water, Sanitation and Hygiene Unit, Ethiopian Public Health Institute, Addis Ababa, Ethiopia; ^119^Department of Psychiatry, Haramaya University, Harar, Ethiopia; ^120^Department of Midwifery, Mekelle University, Mekelle, Ethiopia; ^121^School of Midwifery, University of Gondar, Gondar, Ethiopia; ^122^Department of Pharmacology, Bahir Dar University, Bahir Dar, Ethiopia; ^123^Pharmacy Department, Alkan Health Science, Business and Technology College, Bahir Dar, Ethiopia; ^124^Centre for Fertility and Health, Norwegian Institute of Public Health, Oslo, Norway; ^125^National Data Management Center for Health, Ethiopian Public Health Institute, Addis Ababa, Ethiopia; ^126^Faculty of Health, University of Canberra, Canberra, ACT, Australia; ^127^ International Institute of Global Health, United Nations University, Kuala Lumpur, Malaysia
