# Supplementary material for: National and subnational burden of under-5, infant, and neonatal mortality in Ethiopia, 1990–2019: Findings from the Global Burden of Disease Study 2019
Source: PLOS Glob Public Health. 2023 Jun 21;3(6):e0001471. doi: 10.1371/journal.pgph.0001471 (PMC10284418; doi:10.1371/journal.pgph.0001471)
Supplement: S2 File — (DOC) [file pgph.0001471.s009.DOC]

# **S2 File. GBD 2019 Ethiopia Child Mortality Collaborators Authors’ Contributions**

## Conceptualization

## Gizachew A Tessema, Tezera Moshago Berheto, Amare Deribew, Berihun Assefa Dachew, Yohannes Adama Melaku, Muktar Beshir Ahmed, Nefsu Awoke, Kebede Deribe, Abebaw Alemayehu Desta, Demissie Assegu Fenta, Getachew Tilahun Gessese, Simon I Hay, Worku Misganaw Kebede, Mensur Shafie Mohammed, Getaneh Baye Mulu, Beemnet Tekabe Mulugeta, Christopher J L Murray, Henok Biresaw Netsere, Mengistie Kassahun Tariku, Belay Negash Tefera, Gavin Pereira, Awoke Misganaw, Yohannes Kinfu

## Data Curation

## Gizachew A Tessema, Tezera Moshago Berheto, Yalemzewod Assefa Gelaw, Berihun Assefa Dachew, Mastewal Belayneh Aklil, Nefsu Awoke, Meseret Derbew Molla, Kebede Deribe, Tahir Eyayu, Demissie Assegu Fenta, Daniel Baza Gargamo, Tamirat Getachew, Abraham Tamirat Gizaw, Simon I Hay, Worku Misganaw Kebede, Mohammed Abdurke Kure, Gedefaye Nibret Mihrtie, Beemnet Tekabe Mulugeta, Christopher J L Murray, Mengistie Kassahun Tariku, Belay Negash Tefera, Tewodros Eshete Wonde, Awoke Misganaw

## Formal Analysis

## Gizachew A Tessema, Tezera Moshago Berheto, Berihun Assefa Dachew, Dejene Tsegaye Alem, Nefsu Awoke, Melaku Ashagrie Belete, Natanim Degefu, Msganaw Derese, Demissie Assegu Fenta, Melaku Getachew, Tamirat Getachew, Simon I Hay, Demisu Zenbaba Heyi, Alelign Tasew Jema, Mohammed Abdurke Kure, Beemnet Tekabe Mulugeta, Christopher J L Murray, Mengistie Kassahun Tariku, Tewodros Eshete Wonde, Gavin Pereira, Awoke Misganaw, Yohannes Kinfu

## Funding Acquisition

Fentabil Getnet, Simon I Hay, Mengistie Kassahun Tariku, Tewodros Eshete Wonde, Awoke Misganaw

## Investigation

## Gizachew A Tessema, Tezera Moshago Berheto, Berihun Assefa Dachew, Zohra S Lassi, Nefsu Awoke, Kebede Deribe, Demissie Assegu Fenta, Daniel Baza Gargamo, Getachew Tilahun Gessese, Fentabil Getnet, Abraham Tamirat Gizaw, Gedefaye Nibret Mihrtie, Salahuddin Mohammed, Beemnet Tekabe Mulugeta, Henok Biresaw Netsere, Mengistie Kassahun Tariku, Tewodros Eshete Wonde, Gavin Pereira, Awoke Misganaw, Yohannes Kinfu

## Methodology

Gizachew A Tessema, Tezera Moshago Berheto, Yared Tadesse, Yalemzewod Assefa Gelaw, Amare Deribew, Berihun Assefa Dachew, Mastewal Belayneh Aklil, Abayneh Tadesse Alamer, Daniel Atlaw, Nefsu Awoke, Solomon Shitu Ayen, Melaku Ashagrie Belete, Natanim Degefu, Daniel Berhanie Enyew, Tahir Eyayu, Demissie Assegu Fenta, Zinabu Fentaw, Melaku Getachew, Abraham Tamirat Gizaw, Demisu Zenbaba Heyi, Alelign Tasew Jema, Bekalu Getnet Kassa, Mohammed Abdurke Kure, Maru Mekie, Salahuddin Mohammed, Beemnet Tekabe Mulugeta, Misganu Teshoma Regasa, Bereket Beyene Shashamo, Mengistie Kassahun Tariku, Gebiyaw Wudie Tsegaye, Mandaras Tariku Walde, Tewodros Eshete Wonde, Gavin Pereira, Awoke Misganaw, Yohannes Kinfu

## Project Administration

## Gizachew A Tessema, Tezera Moshago Berheto, Nefsu Awoke, Fentabil Getnet, Simon I Hay, Christopher J L Murray, Mengistie Kassahun Tariku, Tewodros Eshete Wonde, Awoke Misganaw

## Resources

## Gizachew A Tessema, Tezera Moshago Berheto, Fentabil Getnet, Bekalu Getnet Kassa, Mengistie Kassahun Tariku, Tewodros Eshete Wonde

## Software

Gizachew A Tessema, Tezera Moshago Berheto, Mastewal Belayneh Aklil, Nefsu Awoke, Tebabere Moltot Kitaw, Beemnet Tekabe Mulugeta, Mengistie Kassahun Tariku, Tewodros Eshete Wonde

## Supervision

## Melaku Ashagrie Belete, Fentabil Getnet, Simon I Hay, Bekalu Getnet Kassa, Tebabere Moltot Kitaw, Maru Mekie, Belsity Temesgen Meselu, Beemnet Tekabe Mulugeta, Christopher J L Murray, Mengistie Kassahun Tariku, Tewodros Eshete Wonde, Gavin Pereira, Awoke Misganaw, Yohannes Kinfu

## Validation

## Gizachew A Tessema, Tezera Moshago Berheto, Tadesse M Abegaz, Mastewal Belayneh Aklil, Atalel Fentahun Awedew, Nefsu Awoke, Melaku Ashagrie Belete, Belay Boda Abule Bodicha, Chuchu Churko, Abel Fekadu Dadi, Natanim Degefu, Meseret Derbew Molla, Abebaw Alemayehu Desta, Aklilu Endalamaw, Getnet Gedif Engida, Tahir Eyayu, Addis Eyeberu, Zinabu Fentaw, Daniel Baza Gargamo, Getachew Tilahun Gessese, Lemma Getacher, Melaku Getachew, Tamirat Getachew, Fentabil Getnet, Abraham Tamirat Gizaw, Demisu Zenbaba Heyi, Foziya Mohammed Hussien, Bedru Jemal, Bekalu Getnet Kassa, Worku Misganaw Kebede, Tebabere Moltot Kitaw, Mohammed Abdurke Kure, Maru Mekie, Belsity Temesgen Meselu, Gedefaye Nibret Mihrtie, Salahuddin Mohammed, Zewdie Mulissa, Getaneh Baye Mulu, Beemnet Tekabe Mulugeta, Henok Biresaw Netsere, Endalew Gemechu Sendo, Migbar Mekonnen Sibhat, Kasahun Girma Tareke, Mengistie Kassahun Tariku, Belay Negash Tefera, Birhanu Wagaye, Mandaras Tariku Walde, Tewodros Eshete Wonde, Awoke Misganaw

## Visualization

Gizachew A Tessema, Tezera Moshago Berheto, Mastewal Belayneh Aklil, Nefsu Awoke, Melaku Ashagrie Belete, Belay Boda Abule Bodicha, Chuchu Churko, Natanim Degefu, Meseret Derbew Molla, Tahir Eyayu, Addis Eyeberu, Demissie Assegu Fenta, Lemma Getacher, Melaku Getachew, Tamirat Getachew, Abraham Tamirat Gizaw, Demisu Zenbaba Heyi, Foziya Mohammed Hussien, Bekalu Getnet Kassa, Tebabere Moltot Kitaw, Mohammed Abdurke Kure, Maru Mekie, Gedefaye Nibret Mihrtie, Hussen Mohammed, Mensur Shafie Mohammed, Salahuddin Mohammed, Getaneh Baye Mulu, Beemnet Tekabe Mulugeta, Migbar Mekonnen Sibhat, Mengistie Kassahun Tariku, Tewodros Eshete Wonde

## Writing – original draft

Gizachew A Tessema, Tezera Moshago Berheto, Yohannes Adama Melaku, Gavin Pereira, Awoke Misganaw, Yohannes Kinfu

## Writing – review & editing

## Gizachew A Tessema, Tezera Moshago Berheto, Yared Tadesse, Yalemzewod Assefa Gelaw, Amare Deribew, Yohannes Adama Melaku, Zohra S Lassi, Kedir Hussein Abegaz, Tadesse M Abegaz, Kidist Adamu, Mohammed Hussien Adem, Muktar Beshir Ahmed, Gizachew Taddesse Akalu, Mastewal Belayneh Aklil, Addis Aklilu, Abayneh Tadesse Alamer, Dejene Tsegaye Alem, Addisu Alehegn Alemu, Musa Mohammed Ali, Hiwot Amare, Daniel Atlaw, Atalel Fentahun Awedew, Nefsu Awoke, Tewachew Awoke, Tegegn Mulatu Ayana, Solomon Shitu Ayen, Niguss Cherie Bekele, Melaku Ashagrie Belete, Alemshet Yirga Berhie, Belay Boda Abule Bodicha, Chuchu Churko, Abel Fekadu Dadi, Wakgari Binu Daga, Natanim Degefu, Tadesse Mamo Dejene, Getnet Makasha Demeke, Meseret Derbew Molla, Msganaw Derese, Kebede Deribe, Abebaw Alemayehu Desta, Getnet Gedif Engida, Daniel Berhanie Enyew, Tahir Eyayu, Addis Eyeberu, Demissie Assegu Fenta, Zinabu Fentaw, Tomas Y Ferede, Daniel Baza Gargamo, Mesfin Gebrehiwot, Amanuel Tesfay Gebremedhin, Teferi Gebru Gebremeskel, Mathewos Alemu Gebremichael, Getachew Tilahun Gessese, Lemma Getacher, Melaku Getachew, Motuma Erena Getachew, Tamirat Getachew, Alene Geteneh, Fentabil Getnet, Abraham Tamirat Gizaw, Dessalegn Geleta Gobena, Temesgen Worku Gudayu, Abdiwahab Hashi, Simon I Hay, Demisu Zenbaba Heyi, Foziya Mohammed Hussien, Alelign Tasew Jema, Bedru Jemal, Girum Gebremeskel Kanno, Bekalu Getnet Kassa, Getahun Molla Kassa, Adera Debella Kebede, Worku Misganaw Kebede, Getiye Dejenu Kibret, Tebabere Moltot Kitaw, Mohammed Abdurke Kure, Galana Ayana Mamo, Maru Mekie, Bedasa Taye Merga, Belsity Temesgen Meselu, Gedefaye Nibret Mihrtie, Alemu Basazin Mingude, Hussen Mohammed, Mensur Shafie Mohammed, Salahuddin Mohammed, Zewdie Mulissa, Getaneh Baye Mulu, Beemnet Tekabe Mulugeta, Christopher J L Murray, Henok Biresaw Netsere, Misganu Teshoma Regasa, Biniyam Sahiledengle, Endalew Gemechu Sendo, Nigussie Tadesse Sharew, Bereket Beyene Shashamo, Yitagesu Sintayehu, Kasahun Girma Tareke, Mengistie Kassahun Tariku, Belay Negash Tefera, Getaye Worku Tesema, Gebiyaw Wudie Tsegaye, Biruk Shalmeno Tusa, Gebresilasea Gendisha Ukke, Birhanu Wagaye, Mandaras Tariku Walde, Meklit Girma Woldmicheal, Tewodros Eshete Wonde, Ayenew Engida Yismaw, Yazachew Yismaw, Gavin Pereira, Awoke Misganaw, Yohannes Kinfu
