# Supplementary material for: National and subnational burden of under-5, infant, and neonatal mortality in Ethiopia, 1990–2019: Findings from the Global Burden of Disease Study 2019
Source: PLOS Glob Public Health. 2023 Jun 21;3(6):e0001471. doi: 10.1371/journal.pgph.0001471 (PMC10284418; doi:10.1371/journal.pgph.0001471)
Supplement: S3 File — (DOCX) [file pgph.0001471.s010.docx]

**S3 File. GBD 2019 Ethiopia Child Mortality Collaborators list for PubMed indexing**

| **First Names** | **Last Name** |
| --- | --- |
| Gizachew A | Tessema |
| Tezera Moshago | Berheto |
| Berihun Assefa | Dachew |
| Yohannes Adama | Melaku |
| Zohra S | Lassi |
| Kedir Hussein | Abegaz |
| Tadesse M | Abegaz |
| Kidist | Adamu |
| Mohammed Hussien | Adem |
| Muktar Beshir | Ahmed |
| Gizachew Taddesse | Akalu |
| Mastewal Belayneh | Aklil |
| Addis | Aklilu |
| Abayneh Tadesse | Alamer |
| Dejene Tsegaye | Alem |
| Addisu Alehegn | Alemu |
| Musa Mohammed | Ali |
| Hiwot | Amare |
| Daniel | Atlaw |
| Atalel Fentahun | Awedew |
| Nefsu | Awoke |
| Tewachew | Awoke |
| Tegegn Mulatu | Ayana |
| Solomon Shitu | Ayen |
| Niguss Cherie | Bekele |
| Melaku Ashagrie | Belete |
| Alemshet Yirga | Berhie |
| Belay Boda Abule | Bodicha |
| Chuchu | Churko |
| Abel Fekadu | Dadi |
| Wakgari Binu | Daga |
| Natanim | Degefu |
| Tadesse Mamo | Dejene |
| Getnet Makasha | Demeke |
| Meseret | Derbew Molla |
| Msganaw | Derese |
| Kebede | Deribe |
| Amare | Deribew |
| Abebaw Alemayehu | Desta |
| Aklilu | Endalamaw |
| Getnet Gedif | Engida |
| Daniel Berhanie | Enyew |
| Tahir | Eyayu |
| Addis | Eyeberu |
| Demissie Assegu | Fenta |
| Zinabu | Fentaw |
| Tomas Y | Ferede |
| Daniel Baza | Gargamo |
| Mesfin | Gebrehiwot |
| Amanuel Tesfay | Gebremedhin |
| Teferi Gebru | Gebremeskel |
| Mathewos Alemu | Gebremichael |
| Yalemzewod Assefa | Gelaw |
| Getachew Tilahun | Gessese |
| Lemma | Getacher |
| Melaku | Getachew |
| Motuma Erena | Getachew |
| Tamirat | Getachew |
| Alene | Geteneh |
| Fentabil | Getnet |
| Abraham Tamirat | Gizaw |
| Dessalegn Geleta | Gobena |
| Temesgen Worku | Gudayu |
| Abdiwahab | Hashi |
| Simon I | Hay |
| Demisu Zenbaba | Heyi |
| Foziya Mohammed | Hussien |
| Alelign Tasew | Jema |
| Bedru | Jemal |
| Girum Gebremeskel | Kanno |
| Bekalu Getnet | Kassa |
| Getahun Molla | Kassa |
| Adera Debella | Kebede |
| Worku Misganaw | Kebede |
| Getiye Dejenu | Kibret |
| Tebabere Moltot | Kitaw |
| Mohammed Abdurke | Kure |
| Galana Ayana | Mamo |
| Maru | Mekie |
| Bedasa Taye | Merga |
| Belsity Temesgen | Meselu |
| Gedefaye Nibret | Mihrtie |
| Alemu Basazin | Mingude |
| Hussen | Mohammed |
| Mensur Shafie | Mohammed |
| Salahuddin | Mohammed |
| Zewdie | Mulissa |
| Getaneh Baye | Mulu |
| Beemnet Tekabe | Mulugeta |
| Christopher J L | Murray |
| Henok Biresaw | Netsere |
| Misganu Teshoma | Regasa |
| Biniyam | Sahiledengle |
| Endalew Gemechu | Sendo |
| Nigussie Tadesse | Sharew |
| Bereket Beyene | Shashamo |
| Migbar Mekonnen | Sibhat |
| Yitagesu | Sintayehu |
| Yared | Tadesse |
| Kasahun Girma | Tareke |
| Mengistie Kassahun | Tariku |
| Belay Negash | Tefera |
| Getaye Worku | Tesema |
| Gebiyaw Wudie | Tsegaye |
| Biruk Shalmeno | Tusa |
| Gebresilasea Gendisha | Ukke |
| Birhanu | Wagaye |
| Mandaras Tariku | Walde |
| Meklit Girma | Woldmicheal |
| Tewodros Eshete | Wonde |
| Ayenew Engida | Yismaw |
| Yazachew | Yismaw |
| Gavin | Pereira |
| Awoke | Misganaw |
| Yohannes | Kinfu |
